# Overview of Tissue Engineering and Drug Delivery Applications of Reactive Electrospinning and Crosslinking Techniques of Polymeric Nanofibers with Highlights on Their Biocompatibility Testing and Regulatory Aspects

**DOI:** 10.3390/pharmaceutics16010032

**Published:** 2023-12-26

**Authors:** Husam M. Younes, Hana Kadavil, Hesham M. Ismail, Sandi Ali Adib, Somayeh Zamani, Raid G. Alany, Ali A. Al-Kinani

**Affiliations:** 1 Tissue Engineering & Nanopharmaceuticals Research Laboratory (TENRL), Office of Vice President for Research & Graduate Studies, Qatar University, Doha P.O. Box 2713, Qatar; h.kadavil@qu.edu.qa (H.K.); h.elkhadem88@gmail.com (H.M.I.); sandi.aliadib@qu.edu.qa (S.A.A.);; 2 Charles River Laboratories, Montreal, QC H9X 3R3, Canada; 3 Materials Science & Engineering, Cornell University, Ithaca, NY 14853, USA; 4 School of Pharmacy, The University of Auckland, Auckland 1142, New Zealand; r.alany@kingston.ac.uk (R.G.A.); a.alkinani@kingston.ac.uk (A.A.A.-K.); 5 Drug Discovery, Delivery and Patient Care (DDDPC) Theme, School of Life Sciences, Pharmacy and Chemistry, Kingston University London, Kingston upon Thames, London KT2 7LB, UK

**Keywords:** electrospinning, chemical reactive electrospinning, photoreactive electrospinning, three-dimensional nanofibers, tissue engineering, drug delivery, in situ crosslinking, photopolymerization, biocompatibility, FDA regulations

## Abstract

Traditional electrospinning is a promising technique for fabricating nanofibers for tissue engineering and drug delivery applications. The method is highly efficient in producing nanofibers with morphology and porosity similar to the extracellular matrix. Nonetheless, and in many instances, the process has faced several limitations, including weak mechanical strength, large diameter distributions, and scaling-up difficulties of its fabricated electrospun nanofibers. The constraints of the polymer solution’s intrinsic properties are primarily responsible for these limitations. Reactive electrospinning constitutes a novel and modified electrospinning techniques developed to overcome those challenges and improve the properties of the fabricated fibers intended for various biomedical applications. This review mainly addresses reactive electrospinning techniques, a relatively new approach for making in situ or post-crosslinked nanofibers. It provides an overview of and discusses the recent literature about chemical and photoreactive electrospinning, their various techniques, their biomedical applications, and FDA regulatory aspects related to their approval and marketing. Another aspect highlighted in this review is the use of crosslinking and reactive electrospinning techniques to enhance the fabricated nanofibers’ physicochemical and mechanical properties and make them more biocompatible and tailored for advanced intelligent drug delivery and tissue engineering applications.

## 1. Introduction

In the past two decades, the discipline of tissue engineering (TE), which focuses on treating organ failure, has attracted much attention. TE involves repairing and/or substituting damaged tissues or cells by combining cells, biomaterial scaffolds, and suitable bioactive molecules [1]. TE has many advantages compared to traditional treatment, for instance, organ transplantation, which carries a higher risk of immunological reactions and presents significant challenges in identifying suitable donors [2]. Polymeric nanofibers (PNs) are considered excellent matrices for drug delivery and TE applications since they can create an environment favorable for cell maturation and proliferation similar to the extracellular matrix (ECM) [1].

A typical ideal PN scaffold intended for TE applications should possess properties that emulate the natural cellular niche and enable the successful growth and proliferation of seeded cells. First, it should preferably be hydrophilic with a highly porous three-dimensional structure, fostering cellular growth and proliferation akin to the ECM of the cells’ original habitat [3]. Second, it should demonstrate biodegradability and biocompatibility to avoid immune responses and the need for retrieval surgery. Third, it should possess the necessary mechanical properties to provide physical scaffolding for cell maturation. Finally, it should be able to respond to diverse biological signalings [4,5] and enable the transport, delivery, and release of biomolecules essential for cell growth [6].

PNs generally exhibit unique properties, making them highly considered for various biomedical applications. Typically ranging in diameter from 1 to 1000 nanometers, these ultrafine fibers possess a notably high surface-area-to-volume ratio, enabling enhanced cell attachment, proliferation, and differentiation, forming them into a desirable material for use in different biomedical implementations [7]. Additionally, PNs have the potential to be easily fabricated and transformed into various forms, such as scaffolds, membranes, and hydrogels, to meet the particular demands of different biomedical applications [7,8,9]. Moreover, nanofibers can be fabricated from diverse biocompatible and biodegradable polymers, allowing for compatibility with various cell types and tissues.

**Figure 1:**
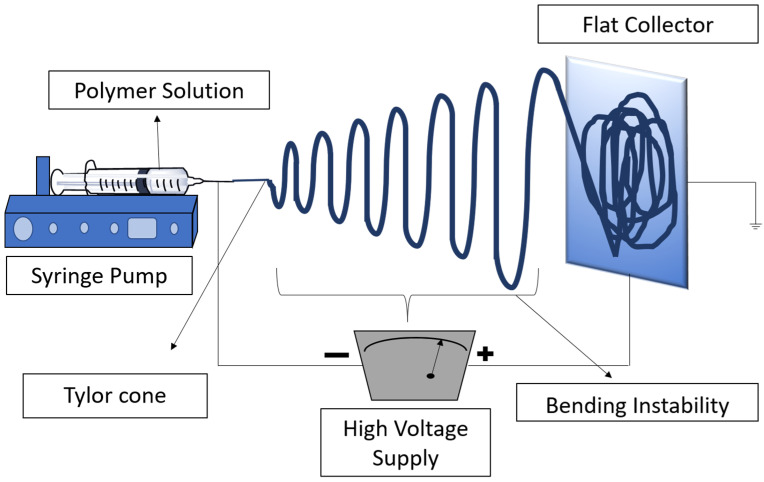
Schematic illustration of traditional electrospinning setup.

PNs can be prepared using several techniques, including but not limited to solution blowing [10,11], self-assembly [12,13], electrospinning (ES) [14], template synthesis [15], and phase separation [16,17]. Among the various approaches, ES is considered the most efficient and widely used method to produce PNs [7]. A typical basic electrospinning setup consists of three parts: (1) a high-voltage power supply, (2) a spinneret (metallic needle), and (3) a grounded metallic collector (Figure 1) [18]. The metallic collectors are generally of three types: stationary flat plates, as shown in the figure; spinning drums; and rotating discs. During the electrospinning process, high-voltage power is applied to the liquid droplets from polymer melt/solution at the tip of the spinneret. When the electrostatic repulsion overcomes surface tension on the droplets, they will elongate into a conical shape jet known as a “Taylor cone.” Fibers with high aspect ratios and diameters that can be precisely controlled are then collected on the grounded metallic collector [19].

Operating, material, and ambient parameters are various variables in the electrospinning technique that tremendously affect the morphology and physicochemical properties of the fabricated electrospun fibers (ESFs). The operating parameters consist of the applied voltage or electric field, the flow rate of polymer melt/solution, the distance between the tip of the metallic needle and collector, and the diameter of the needle. A small change in those parameters can lead to a significant change in the fabricated fiber morphology. The material parameters include but are not limited to polymer concentration, viscosity, surface tension, and conductivity. On the other hand, ambient parameters, such as temperature and humidity, which are related to the surrounding environment of the electrospinning jet, may alter the electrospinning process and fiber morphology [20].

Although conventional electrospinning is reported to have many pros and wide applications, it has several limitations, including weak mechanical strength, large diameter distributions, and scaling-up difficulties of the produced electrospun nanofibrous scaffolds. The constraints of the polymer solution’s intrinsic properties, including viscosity, surface tension, and conductivity, are primarily responsible for these limitations [21]. Due to all these challenges, other novel and modified electrospinning techniques, such as reactive electrospinning, are needed to overcome those challenges and improve the properties of fabricated fibers intended for various applications.

Reactive electrospinning (RES) is a versatile method for creating functional fibers with various characteristics tailored to specific biomedical applications. It has recently gained much attention as it can fabricate fibers with tunable mechanical and functional properties by applying different crosslinking strategies and incorporating various chemical or biochemical reactions into the spinning process. These crosslinking strategies are widely used to amplify polymers’ chemical stability and mechanical properties in support structures for various biomedical applications. The RES can be carried out during fiber generation (in situ) or after it (post-), resulting in improved structural integrity and durability of the material. For instance, hydrophilic polymers, more prone to mechanical degradation when in touch with the biological environment, pose a significant challenge for applications such as cell seeding, in vivo implantation, and transfer from cell culture [21]. Research in mechanobiology has also highlighted the importance of simulating the scaffold’s elasticity with that of the host tissue to reduce immunogenic reactions and, subsequently, any local inflammatory responses during implantation [22]. On the other hand, by altering the surface characteristics of the polymer by adding specific reactive groups, sustained release of hydrophobic/hydrophilic medicines from a scaffold with opposite polarity can be accomplished. This method enables the creation of a scaffold with desired drug-release properties by customizing the hydrophilicity of the polymer.

This review provides a comprehensive overview of RES, including recent reports of electrospun fibers (ESF) generated by various RES techniques and crosslinking strategies. It delves into chemical and photoreactive electrospinning, including reactive groups and crosslinking agents. Additionally, this article explores the potential biomedical applications of RES in TE and drug delivery. To our knowledge, this constitutes the first focused review of this area. It also provides an overview of the studies and research conducted on reactive electrospinning, the challenges encountered, and their possible solutions. It also provides an overview of the methods of conducting in vitro and in vivo biocompatibility and degradability studies of electrospun-nanofiber-based devices. It also highlights the regulatory aspects that govern their development and marketing approvals.

## 2. Reactive Electrospinning (RES)

Since 2005, when Kim et al. introduced the concept of RES [22], the technique has become increasingly popular in improving the mechanical robustness of polymer nanofibers. This technique incorporates an in situ crosslinking step throughout or after the fiber preparation. By doing so, the resulting polymers can strike a balance between their mechanical and water stability with time, resulting in a material with improved properties [8,23]. RES can be classified into two distinct categories: chemical reactive electrospinning (CRES) and photoreactive electrospinning (PRES). In CRES, crosslinking is initiated by chemical means, while PRES involves photoinitiated crosslinking. This approach enables the customization of the functional properties of the polymer fibers and provides a versatile tool for the design and synthesis of advanced materials with tailored functionality.

### 2.1. Chemical Reactive Electrospinning (CRES)

The idea behind CRES is to use reactive chemical substances or functional groups to alter the surface or characteristics of the nanofibers while they are being electrospun. Typically, the reactive chemical is added to a polymer solution, which is then charged before being ejected via a spinneret to create a fiber. During electrospinning, a chemical reaction between the reactive chemical in the solution and the fiber surface results in a crosslinking of the scaffold suitable for intended biomedical applications. Several in situ crosslinking strategies were utilized, and variations in the electrospinning setup were tailored depending on the kinetics of the crosslinking reaction.

#### 2.1.1. Designs and Setups

**Figure 2:**
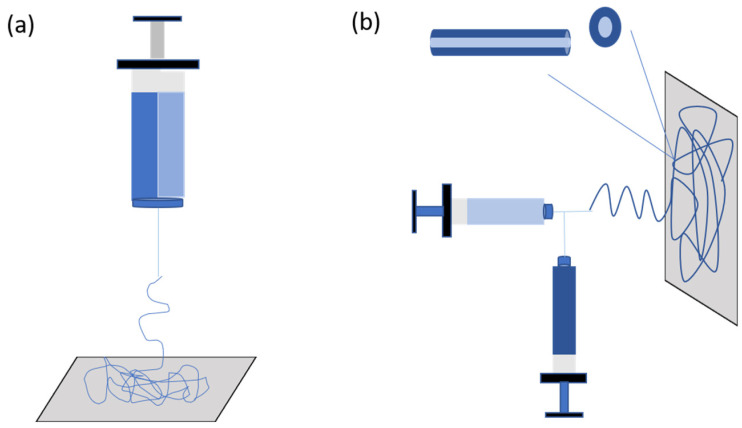
A sketch illustrating various setups of CRES: (**a**) double-barrel electrospinning and (**b**) coaxial electrospinning.

Coordination of nanofiber ejection and crosslinking is crucial while using CRES techniques, mainly when both co-occur to ensure the success of the ES setup. This requires coordinating the desired electrospinning setup with the available time frame, which is determined by the crosslinking chemistry’s kinetics. A critical level of crosslinking in the spinning solution can affect the creation of a stable polymer jet, which can affect the formation of fibers. For instance, if crosslinking occurs during the electrospinning process, it can cause a change in the solution viscosity and result in variations in fiber diameter, as seen in the case of glyoxal and gelatin [24]. Various electrospinning setups have been developed to enable the crosslinking of polymers, including selecting the syringe and needle shape based on the crosslinking kinetics. For instance, if the reaction is spontaneous, double-barrel-syringe and coaxial-needle setups may be employed [25]. Additionally, direct mixing of the crosslinking agent and polymer can be adequate when the crosslinking kinetics time equals the electrospinning time frame. These variations in electrospinning setups offer a flexible and versatile approach for synthesizing polymeric fibers with tailored crosslinking and physicochemical properties, suitable for various biomedical applications (Figure 2).

##### Double Barrel/Dual Syringe

**Figure 3:**
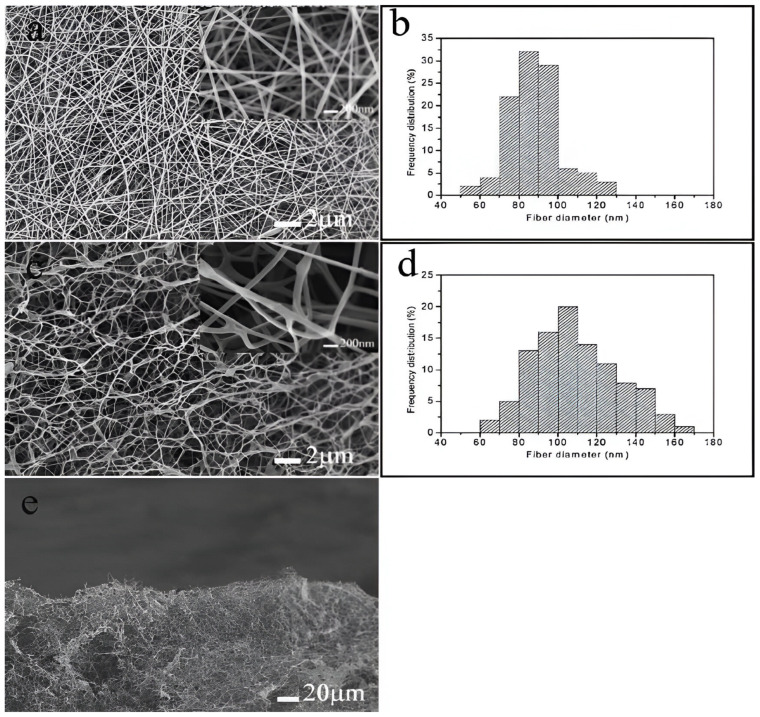
(**a**) SEM micrograph of HA-DTPH/PEO-blend scaffolds before PEO extraction, and (**b**) the corresponding histogram of the fiber diameter distribution. (**c**) SEM micrograph of HA-DTPH nanofibrous scaffolds after PEO extraction, and (**d**) the corresponding histogram of the fiber diameter distribution. (**e**) SEM micrograph of the cross-sectional view of electrospun HA-DTPH nanofibrous scaffolds. (Insets are the high magnification SEM micrographs, scale bar = 200 nm) (Reproduced from reference [26] with permission from Wiley).

To address the challenges associated with variations in viscosity during the electrospinning process and the need to accommodate the swift chemical reactions like aldehyde–hydrazine or blocked iso-cyanate–amine, a feasible solution is to use a double-barrel syringe to avoid the faster crosslinking process. This technique allows for the separation of fluids before spinning, thus enabling more effective control over the crosslinking process. Ji et al. prepared water-stable low-molecular-weight hyaluronic acid (HA) nanofibers using a double-barrel syringe. The modified low-molecular-weight HA derivative called 3,3′-Dithiobis(propanoic dihydrazide) (HA-DTPH) was added to the procedure, and it was crosslinked utilizing the homo-bifunctional crosslinker polyethylene glycol diacrylate (PEGDA) to create nanofibers with adjustable crosslinking densities (Figure 3). Moreover, a dual-syringe mixing method was utilized in another study during the electrospinning procedure in which modified HA and a crosslinker were incorporated in separate syringes to prevent clogging. The low-viscous HA solution was combined with PEO to improve the solution viscosity and was then selectively removed by soaking in water. Following a test for biocompatibility, 3T3 fibroblast cells matured into fibers and formed a 3D dendritic network. This technique offered the benefit of tuning the crosslinking degree and, as a result, the scaffold’s material properties [26].

##### Direct Mixing

When the crosslinking rate is the same as the electrospinning time, the solution can be directly mixed in one pot. Kosha et al. studied the one-pot in situ crosslinking of chitosan/polyvinyl alcohol (PVA) with glyoxal as a crosslinker incorporated with halloysite nanotubes (HNT) using a step-by-step direct mixing approach, followed by immediate electrospinning without further treatment for stabilization. It was verified that an acetal bond existed between the glyoxal and hydroxyl groups of PVA and chitosan, and this reduced the contact angle with water, increasing hydrophilicity and enhancing fibroblast cell attachment. The biocompatibility and mechanical strength of chitosan/PVA nanofibers were enhanced by the presence of HNT [27].

##### Coaxial Electrospinning

In CRES, the coaxial setup results in the fabrication of core–shell-structured fibers. This design is highly beneficial because it allows for the simultaneous deposition of two or more different materials in a single continuous fiber, resulting in unique material properties and improved functionality via diverse polymer–crosslinker combinations. Numerous research studies have utilized the coaxial electrospinning method, predominantly incorporating crosslinkers into the inner region and polymer as the outer layer of the needle. Notably, a study conducted by Gualandi et al. produced gelatin nanofibers of exceptional crosslinking through coaxial electrospinning, utilizing a combination of gelatin and natural crosslinker genipin solutions. After thermal treatment and rinsing in ethanol and phosphate buffer saline (PBS), the fabricated nanofibers exhibited improved mechanical properties and maintained their morphology in aqueous solutions [28]. Another study was carried out by Molnar et al. about polymer fibers based on poly(aspartic acid) produced by utilizing poly(succinimide) in the outer layer and hexane diamine crosslinker in the inner core [29]. The fabricated ESFs were pH sensitive and underwent shrinking in an acidic pH of 4.2, where the carboxylic group was protonated while deprotonation of carboxylic groups in an alkaline medium rendered higher hydrophilicity and swelling of the fibers. The large pore size in these ESFs enabled cell migration inside the matrix, and the reversible pH responsiveness was useful in drug delivery and TE applications. The study, however, did not include any information regarding the fibers’ mechanical properties, which play an essential role in TE applications.

#### 2.1.2. Crosslinkers and Crosslinking Strategies in CRES

The fabrication of appropriate scaffolds for biomedical applications has been the subject of numerous investigations aimed at exploring diverse crosslinking strategies. Crosslinking of electrospun scaffolds involves connecting functional groups that are unveiled from scaffolds. The crosslinking agents chemically bond more than two reactive ends to link polymer chains in the scaffold to exhibit optimal physical stability when it is intended to be placed in the body. The diversity in crosslinkers and crosslinking schemes helps preserve the nanofibrous features resembling native ECMs. Moreover, The use of additional crosslinking can assist in preventing the breakdown of nanofibers and improve their mechanical properties, allowing for electrospun scaffolds that are more durable and capable of tolerating a variety of bioenvironmental challenges, including changes in pH, exposure to enzymes, and biomechanical stresses inside the biological environment.

**Figure 4:**
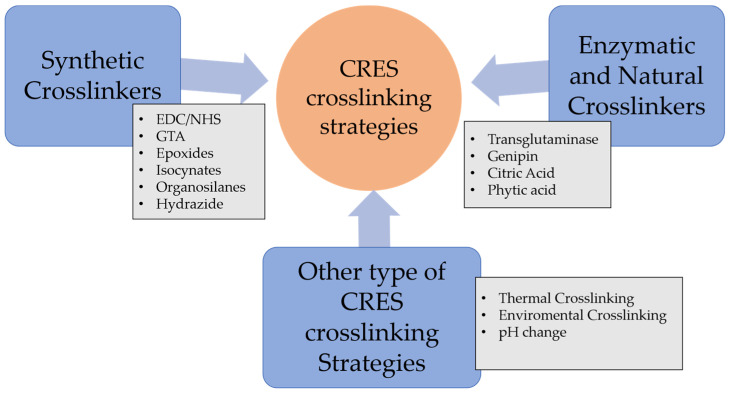
Illustrative overview of CRES crosslinking strategies explored in this review.

Various crosslinking strategies are employed in CRES, including zero-length and non-zero-length crosslinking. Zero-length crosslinking occurs when the crosslinker acts as a catalyst in a covalent reaction, while non-zero-length crosslinking involves the crosslinker becoming part of the reaction itself. In addition to the chemical and natural crosslinkers, CRES employs various crosslinking strategies, including thermal, environmental, and pH-induced crosslinking (Figure 4). This diverse array of strategies and crosslinkers enhances the versatility of the CRES process, allowing for the creation of functional scaffolds tailored to the specific requirements of various tissue engineering applications.

##### Synthetic Crosslinkers

Various crosslinkers are employed in CRES, including synthetic and natural varieties. Common chemical crosslinkers utilized in CRES comprise a combination of N-ethyl-N′-(3-(dimethylamino) propyl) carbodiimide (EDC) and N-hydroxysuccinimide (NHS), as well as glutaraldehyde [30,31], isocyanateb [32], hydrazine compounds [33], and epoxides [34]. In addition to these chemical crosslinkers, natural crosslinkers such as enzymes, genipin, glyoxal, citric acid, and phytic acid are also utilized in CRES and will be covered later in this review.

##### N-ethyl-N-(3-(dimethylamino) propyl) Carbodiimide (EDC) and N-Hydroxysuccinimide (NHS)

N-ethyl-N′-(3-(dimethylamino) propyl) carbodiimide (EDC) catalyzes the crosslinking without taking part in the polymer network. The EDC is the most commonly used with the absence or presence of N-hydroxysuccinimide (NHS). The carboimmide chemistry of EDC has been extensively used for diverse natural polymers such as gelatin, collagen, and fibrinogen [35,36,37,38,39]. The hydrophilic EDC stimulates the protein’s side groups, thereby enabling the formation of stable bonds with other side groups, such as the ester-bond between the carboxyl and hydroxyl group of biobased polymers [40]. It was reported that an active O-acylisourea derivative was created between the EDC and carboxyl group of the polymer fiber. This was followed by a powerful nucleophilic substitution reaction involving primary amines. When not involved in bond formation, EDC is converted to nontoxic N-substituted urea [41,42]. The effectiveness of the EDC procedure on the carboxyl group has been improved by a technique that uses either N-hydroxysuccinimide or sulfo-N-hydroxysuccinimide. Steady NHS esters are produced when this method is compared to O-acylisourea [40]. The crosslinking method with EDC and NHS provides several benefits, including high efficiency of conversion, mild reaction protocols, and the retention of biocompatibility, such as in gelatin [43]. Numerous studies investigated the characterization and fine-tuning of hydrogels, which have numerous uses in tissue engineering and regenerative medicine [44,45,46], made through the EDC/NHS crosslinking process [47,48,49].

Hajiabbas et al. conducted in situ gelatin crosslinking using the EDC/NHS crosslinking method [50]. The aim was to improve the fiber morphology and mechanical stability of electrospun gelatin (ESG) scaffolds through on-site crosslinking using a novel ethanol PBS solvent system. The effect of solution parameters on fiber diameter was investigated using response surface methodology (RSM), and an ideal setting for producing smooth fibers with a desired diameter was identified. The evaluation of mechanical characteristics and cell toxicity followed. The outcomes demonstrated that by examining the impact of solution factors on the size and morphology of fibers, RSM helped obtain smooth nanofibers. The mechanical properties of ESG scaffolds were significantly improved despite the fact that there were no significant changes to their chemical structure, demonstrating the effectiveness of the novel solvent system and in situ crosslinking technique for the preparation of G fibrous scaffolds. Another research study used EDC as a crosslinker to in situ crosslink chitson (CS) and recombinant gelatin in the absence of (NHS). Surprisingly, the scaffolds’ nanofibrous morphology was maintained entirely, unlike traditional post-crosslinking techniques, where the scaffold’s porosity was reduced by one-third. The scaffolds also had porosities above 60% and a high surface-to-volume ratio, which resulted in considerably higher water uptake (1314% instead of 927%) [51]. These phenomena made biomaterial scaffolds suitable for tissue engineering applications.

##### Glutaraldehyde (GTA)

The most popular crosslinker in CRES is glutaraldehyde (GTA), which typically becomes part of the polymeric network. It is a bifunctional substance with highly reactive aldehyde groups that can bond with thiols, phenols, hydroxyl, imidazole, and amine groups [52]. It functions as a ubiquitous crosslinking agent for proteins by chemically reacting with the glutaraldehyde group and the protein’s amine groups of hydroxylysine or lysine [53]. Including GTA (0.5 M) in the in situ crosslinking increases the toughness of the PVA electrospun nanofibers. The tensile strength increases significantly, by six times, and the scaffold’s elongation is notable [54]. The GTA also modifies the electrospun scaffold’s surface characteristics. This alteration is ascribed to the decrease in hydrophilic groups in gelatin nanofibers following treatment, which leads to improved hydrolytic resistance to water in GTA-cured gelatin nanofibers compared to untreated ones [55]. However, as reported in numerous studies [56,57], the toxicity of glutaraldehyde presents significant health risks, including chronic bronchitis and potential genetic activity. Alternative crosslinking chemicals are thus required, ideally with minimal toxicity and effective crosslinking properties. In the crosslinking of chitosan/PVA nanofibers [27] and gelatin [58], glyoxal was found to be a GTA substitute that exhibits lower cytotoxicity.

##### Epoxides

The three-membered ring structure of epoxides makes them highly reactive toward nucleophiles like hydroxyls, amines, and thiols, forming ethers and substituting amines. Epoxy coupling is another method for CRES. In one study, gelatin was prepared and in situ crosslinked with 1,4-butanediol diglycidyl ether (BDDGE) at various ratios such as 2 wt%, 4 wt%, and 6 wt% and incubation periods of 24, 48, and 72 h at 37 °C. By adjusting the quantity of crosslinker and incubation time, the degree of crosslinking was changed, allowing for control over the diameter of the fiber and mechanical characteristics. BDDGE concentrations of 4% and 6% produced scaffolds with steady diameters of 339 nm and 276 nm after being incubated for 72 h. However, the best pair of mechanical properties was offered by 4% BDDGE [34]. The meshes fabricated showed no toxic effects on fibroblasts and encouraged their adhesion, proliferation, and production of new ECMs, demonstrating the promise of this approach for the engineering of skin tissue. [59].

##### Isocyanates

Isocyanate coupling represents a promising technique for creating urea and urethane bridges via crosslinking polymers containing amino and hydroxyl groups. In a study that enhanced the preservation of ESF morphology postimplantation, a novel approach was employed involving in situ gelatin crosslinking with a 1,6-hexamethylene diisocyanate (HDMI) as a crosslinker. This technique involved the use of a double-barrel syringe. In contrast to unlinked meshes that lost their fibrous structure within 24 h, those fiber meshes that underwent in situ crosslinking were able to maintain their form for a week when submerged in water at a temperature of 37 °C. The research also looked into how crosslinker ratio, crosslinking level, tensile mechanical characteristics, and degradation rates related to one another. It was found that raising the crosslinker ratio resulted in a regulated rise in fiber retention and crosslinking intensity. The crosslinker ratio improved the scaffold’s initial stiffness and tensile strength [60].

##### Organosilanes

The use of multifunctional organosilanes, such as tetraethylorthosilicate (TEOS), which undergo a reaction with hydroxyl groups to form siloxane bridges that connect the crosslinker and polymer substrate through a sol-gel reaction, is another alternative technique for CRES [61]. This procedure needs a catalytic quantity of acids or bases to start the reaction. PVA was also used as it includes a lot of hydroxyl functionalities, especially at elevated deacetylation degrees. Furthermore, this technique facilitates the utilization of eco-friendly solvents such as water and EtOH. One study thoroughly investigated the effects of TEOS concentration, the silica–PVA ratio, the mixture of silica precursors’ aging duration, and the impact of solution viscosity on fiber structure. The outcomes showed a composition frame that produced composite nanofibers with defects as tiny as 150 nm in diameter. Despite PVA’s solubility in water, the hybrid strands held up well when left in water for an extended period. The presence of the Si-O-C peak in the spectral profiles of each of the hybrid samples, which was revealed by FTIR analysis, suggested that PVA and silica formed a bond and that the -OH peak of PVA vanished. These thermally robust and comparatively inert silica-based crosslinked PVA nanofibers could increase the range of applications for these materials in various technologies [62].

##### Hydrazide

This crosslinker is traditionally used to link aldehyde or ketone groups with other molecules. Hydrazone bonds can develop with this chemistry at room temperature, which is appropriate for CRES. In a study involving hydrazides, Hoare et al. invented the use of easily water-soluble polymer precursors derived from PEG. In their research, poly(oligo(ethylene glycol) methyl ether methacrylate (POEGMA) modified with aldehydes was kept apart from hydrazide before being mixed in situ using double-barrel needles. The resulting nanofibers maintained a textured nanofibrous framework in the expanded state and ranged in average diameter from 0.34 ± 0.08 μm to 1.33 ± 0.20 μm. After a few minutes, the structures showed a high degree of swelling of 91% and could maintain an elastic modulus of 2.1 kPa for at least 40 repetitions. According to the authors, the hydrogel may have “smart” thermosensitivity based on the side-chain size of the oligo-ethylene glycol methacrylate monomer, enabling dynamic tuning of the interaction between cells and the gel [63].

##### Enzymatic and Natural Crosslinkers

In CRES, using toxic crosslinkers like glutaraldehyde, epoxides, and isocyanates can lead to residual crosslinkers, which may cause increased cytotoxicity and affect the transplantation of the scaffold in TE applications. Hence, using enzymatic crosslinking and natural crosslinkers has garnered significant attention recently. This can be attributed to the feasibility of producing hydrogels supporting cell growth and proliferation through an enzymatic-driven crosslinking reaction. Notably, this approach offers enhanced cytocompatibility and can be conducted within a cell-friendly environment. This approach has been highlighted in several studies [64,65], exploring their potential in TE and regenerative medicine. Transglutaminase, genipin, and citric acid were among the most extensively and potentially studied under this category and covered in this review.

In developing enzymatic reactive electrospun fibers, transglutaminase has shown promise as an enzyme capable of catalyzing the crosslinking reaction between glutamine and lysine residuals. To date, only one study has reported gelatin crosslinking using this method. The research used premixed gelatin with transglutaminase as the shell and a coaxial needle to spin PVA as the core flow. The resultant scaffold had a relatively smooth surface and 270 nm as an average diameter, but no data were reported on its mechanical properties or biodegradation profile in the water. Nonetheless, the study highlighted the potential of transglutaminase in creating enzymatically crosslinked electrospun fibers, but further studies are needed to dictate the mechanical and biodegradation characteristics of such fibers [66].

Genipin (GP) is a crosslinker derived from the gardenia fruit and exhibits a low level of toxicity [67]. This substance is derived from geniposides, one of the parent compounds found in Gardenia Jasminoides Ellis fruit. When it reacts with glycine, leucine, glutamic acids, and other amino acids, it rapidly turns into a blue pigment through a spontaneous process [68]. A recently published study established an eco-friendly reduction procedure to produce water-soluble chitosan/polyvinyl alcohol (WSCHT/PVA) nanofibers at varying mass ratios using genipin as a crosslinking agent. The study utilized multiple analytical techniques to assess the nanofibers’ structure, morphology, and properties. Results indicated that the 20/80 blend ratio exhibited the most optimal and uniform morphology when compared to the other ratios. Additionally, the nanofiber membranes demonstrated hydrophilic behavior, as indicated by contact angle measurements. The crosslinked WSCHT/PVA nanofibers displayed consistent ejection of drug hesperetin for up to 12 h and showed sufficient adsorption rate in RB5 dye measurements. The study suggests that the prepared scaffold may have potential applications in reducing the negative impact of environmentally toxic chemicals. Thus, the findings serve as a significant development in green nanotechnology [69].

Citric acid, a biogenic tricarboxylic acid present in citrus fruits, can be used as a natural crosslinker to develop polyvinyl alcohol (PVA)-based electrospun scaffolds that are suitable for wound care. Citric acid and glyoxal were used to create chemical crosslinks, slowing the rate at which the structure degraded in a water-based environment [70]. Additionally, cellulose nanoparticles were used to improve the mechanical qualities of the finished electrospun fibers [71].

The use of phytic acid (PA), a natural crosslinker that also has antioxidant and anticancer characteristics, was also reported to enhance the mechanical properties of gelatin (Ge) and poly (ε-caprolactone) (PCL) scaffolds. According to the study’s findings, Young’s modulus and elongation dramatically increased at the recommended level of PA (7.5%) without any adverse side effects [72].

Table 1 lists a summary of the commonly used synthetic and natural chemical crosslinkers with details pertaining to their chemical structure and cytocompatibility.

#### 2.1.3. Other Crosslinking Strategies in CRES

##### Thermal Crosslinking

Thermal crosslinking is a process that entails the use of heat to induce a reaction between polymer chains and create crosslinks. In the context of CRES, thermal crosslinking is often employed to make robust the mechanical characteristics of produced scaffolds. This is achieved by subjecting the electrospun fibers to a controlled amount of heat, which causes the polymer chains to react and form crosslinks. Compared to other types of crosslinking, such as photoinduced or chemical crosslinking, thermal crosslinking may offer greater flexibility in its application. For example, photoinduced crosslinking requires specific wavelengths of light to activate the crosslinking process, which can limit its effectiveness in specific applications. Similarly, chemical crosslinking may require the use of toxic chemicals, which can pose health and safety risks. In contrast, thermal crosslinking is an efficient and straightforward approach that could be easily integrated into the electrospinning process without requiring specialized equipment or chemicals.

One main disadvantage is that the high temperatures required for thermal crosslinking can damage or denature sensitive biomolecules such as proteins, enzymes, or living cells, which may compromise their functionality or viability. Additionally, the crosslinking process may not be uniform, resulting in areas of varying mechanical properties within the same material. This can be problematic in biomedical applications such as tissue engineering or drug delivery, where consistent and predictable mechanical properties are essential. Furthermore, the use of thermal energy can also change the morphology of the electrospun fibers, which can affect their biocompatibility and suitability for use in vivo. Therefore, it is crucial to consider the potential drawbacks of thermal crosslinking in biomedical applications carefully and optimize the conditions to minimize any adverse effects.

In a study conducted by Niu et al., the coaxial electrospinning approach was employed, along with thermal crosslinking, to produce scaffolds with high elasticity and tensile strength. The core polymer was polydimethylsiloxane (PDMS), cured by thermal treatment on a hot plate during electrospinning. The PDMS core was encapsulated in a polyvinyl pyrrolidone (PVP) shell in the coaxial setup, and the PVP coat was subsequently leached out, leaving behind the crosslinked PDMS fibers. The produced scaffolds showed an exceptionally high elasticity [75].

##### Environmental Crosslinking

Environmental crosslinking is another technique employed to crosslink polyamide-based gel scaffolds rapidly using oxygen in the atmosphere. In a study by Molnar et al., the researchers utilized cysteamine moieties grafted on polysuccinimide dissolved in dimethylformamide (DMF) for electrospinning [76]. The oxygen in the air reacted with the sulfide moieties in the polymer solution to induce a fast crosslinking reaction and form the desired electrospun fibers (ESF). The authors explained the chemical reactions and mechanisms involved in the reaction and fiber formation. Scanning electron microscopy (SEM) revealed the formation of ESFs, but the SEM analysis of the crosslinked replica was not conducted. Despite this limitation, the technique introduced by the researchers offered a novel method to improve the molecules’ diffusion into the scaffolds. However, atomic force microscopy showed that the fibrous framework was demolished upon wetting, as the fibers inflated and merged upon drying due to their high hydrophilicity [76]. Jedlovszky-Hajdu, Molnar, and their coworkers continued this work by preparing magnetic, hyperthermic fibers [77]. Using the same polymer and crosslinking method (cysteine sulfur groups crosslinked to disulfide bond), they observed that when the magnetic nanoparticle loading was low, 2D ESF was obtained; however, by increasing the percentage of the nanoparticles, the resulting ESFs were a 3D scaffold. Such magnetic ESFs provided a higher surface area for cell attachment. No biocompatibility was performed in this work, and the dissolution test was only carried out for the non-crosslinked fibers [77]. It would have been much more beneficial and informative if the study compared the dissolution of the non-crosslinked fibers and cysteine-crosslinked ESFs.

##### pH Change

Recent reports suggested that pH changes during electrospinning can be exploited to promote fiber formation. In one study, a green solvent, acetic acid/water, was utilized to dissolve lignin/chitosan/PEO to produce electrospun fibers through polyelectrolyte complexation facilitated by the pH change during solvent evaporation [78]. The fibrous mats produced were soaked in water to eliminate the PEO, which enhanced the bond strength of the polyelectrolyte complexation and increased thermal stability. However, the authors did not compare the electrospinning process with and without PEO. The fibrous structure’s SEM images revealed some branching and merging spots where several fibers formed a single, thick fiber. However, using environmentally friendly solvents in this strategy offered future research opportunities to examine potential uses.

#### 2.1.4. In Situ vs. Postchemical Crosslinking

Studies have investigated the benefits of using in situ crosslinking versus post-crosslinking techniques for electrospun fibrous mats [79,80]. The post-crosslinking process may cause uneven crosslinking, leading to the loss of the scaffold’s porous morphology and the presence of harmful crosslinking agents, making most of them unsuitable for biomedical applications [79,80]. To prove the advantages of in situ and real-time crosslinking on electrospun collagen scaffolds, Meng et al. performed a study that contrasted the two approaches using a mixture of EDC/NHS crosslinkers [81]. The study demonstrated that in situ crosslinking prevented scaffold shrinking and maintained its porous structure after immersion in water. Upon mechanical testing, the in situ crosslinked scaffolds exhibited comparable mechanical properties between their dry and hydrated states, yielding satisfactory results. Despite that, further mechanical testing between in situ crosslinked and post-crosslinked scaffolds and in vitro studies for cell or drug release are needed to assess their potential for biomedical applications. Although the one-step in situ crosslinking process is delicate and influenced by humidity and crosslinker ratios, it offers several advantages over post-crosslinking techniques, including but not limited to using cost-effective materials, being easy to automate, maintaining an excellent spatial distribution in the polymer matrix, and having the ability to integrate with many other heating and curing methods, resulting in better physicochemical and mechanical properties of the fabricated polymeric fibers [82].

Several studies have also covered in situ crosslinking with post-crosslinking techniques using synthetic materials for TE uses. In research using GTA as the crosslinker, Yuan et al. compared pure, in situ crosslinked, and post-crosslinked PVA fibers [83]. The optimal material attributes for ES were ascertained using 12% pure PVA as a reference. The fiber shape, water resistance, mechanical characteristics, and thermal stability of the in situ thermal/GTA crosslinked fibers were contrasted with those of the post-crosslinked fibers [83,84]. The outcomes showed that after being submerged in water for 24 h, the scaffolds of PVA crosslinked in situ possessed a greater elastic modulus while maintaining filamentous morphology. Additionally, compared to post-crosslinked fibers, in situ crosslinked fibers had greater thermal stability. Their use in long-lasting distribution may be constrained by the fact that the weight loss associated with prolonged immersion in water for longer than 24 h was not assessed. According to the research, in situ crosslinked fibers have improved mechanical properties, as evidenced by a rise in Young’s modulus compared to post-crosslinked fibers. According to SEM findings, less than 10% PVA concentrations produced nonbeaded fibers, while concentrations below 12% produced beaded fibers [85]. Finally, additional research is necessary before using these scaffolds in TE applications, especially when using GTA, which has cell toxicity and involves heat treatment, which may impede the integration and delivery of biologically active compounds and proteins.

Another study used a single-fluid electrospinning technique to create nanofibers from mixtures of sodium caseinate (SC) and polyvinyl alcohol (PVA). The main topics of the research work were the production and examination of nanofibers made from various PVA/SC mixing ratios. After careful evaluation, the PVA/SC (70/30, *v*/*v*) mixture was chosen for its bead-free and homogeneous surface qualities. The impact of in situ and post-crosslinking techniques on the properties of nanofibers were compared in the research using GTA. According to the results, the in situ crosslinking technique enhanced thermal properties more than other methods. A ZnO nanoparticle was incorporated within the scaffold. The results demonstrated that the nanofibers’ elongation values significantly increased by including ZnO nanoparticles. However, nonhomogeneous mat surfaces were noted because of the significant ZnO nanoparticle aggregation. The research concluded that the low cell viability of ZnO-doped nanofibers precludes their use as wound dressings [86].

### 2.2. Photoreactive Electrospinning (PRES)

**Figure 5:**
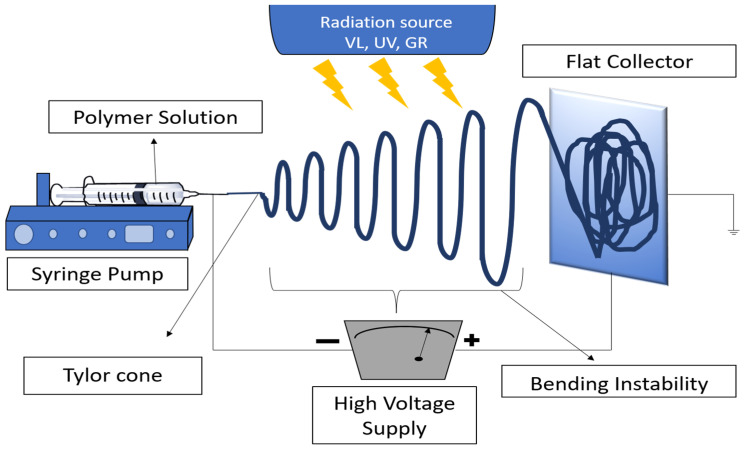
Schematic diagram depicting the PRES setup using different radiation sources. (VL, visible light; UV, ultraviolet light; and GA, gamma rays).

Using PRES has been demonstrated to be a very successful and viable solution for overcoming numerous limitations encountered during CRES procedures. Notably, eliminating cytotoxic crosslinkers such as glutaraldehyde and the difficulties associated with their residue removal have posed a considerable challenge. Therefore, producing photo-crosslinked fibers to circumvent these issues has emerged as an area of interest. Compared to post-crosslinking techniques, which can result in uneven crosslinking on the outside and inside of the fibers, in situ photo-crosslinking provides a more evenly distributed crosslinking throughout the fibers (Figure 5). Subsequently, the mechanical behavior of the resulting electrospun scaffolds is affected by this. Various strategies have been investigated to maximize the advantages of PRES concerning the planned application.

In PRES, the polymer solution used for electrospinning is supplemented with a photosensitive substance or a photoinitiator. After that, the polymer solution is subjected to electrospinning onto a collector substrate and to a particular wavelength of light. The polymer chains crosslink and create a three-dimensional network structure because of the photochemical reaction of light irradiation, creating a fibrous mat with improved mechanical qualities and stability. According to the underlying theory of PRES, photoinitiators produce reactive intermediates or free radicals that can start the crosslinking reaction and change the characteristics of the polymer. The advantages of this method include the ability to regulate the nanofibers’ spatial distribution and degree of crosslinking, which results in better mechanical stability and characteristics. Potential uses for PRES include drug delivery, tissue engineering, wound healing, and many other biomedical disciplines. The reader is advised to refer to the following comprehensive reference on photopolymerization and photoinduced crosslinking technology utilization in tissue engineering and drug delivery [87].

#### 2.2.1. PRES Design and Setups

PRES design and setups are the same as general electrospinning but with the addition of a photo energy source. PRES has swift crosslinking kinetics, which are compatible with the electrospinning time frame [88] making crosslinking within the framework quick, even, and homogeneous. In addition, the exclusion of the use of toxic chemical crosslinkers and sometimes organic solvents creates more biocompatible structures, which makes it possible to load proteins and growth factors into the nanofibers. Finally, the crosslinking process is instigated by applying ultraviolet (UV), gamma radiation (GR), or visible light (VL).

#### 2.2.2. Energy Sources of PRES

PRES requires an efficient energy source that allows photo-crosslinking to occur quickly while the jet flows to the collector. Additionally, the energy supply must be reasonably secure. Due to its low intensity and the longer times needed to achieve the appropriate degree of crosslinking, VL is less used. As a result of meeting the efficient energy requirements, UV light was chosen as the most favored crosslinking source in most published reports despite the better safety, compatibility, and some unique advantages VL offers. On the other hand, due to safety concerns, only a few studies used gamma radiation as a crosslinking source [89].

##### Visible Light

The implementation of visible-light-induced crosslinking is poised to solve many of the challenges currently faced in advancing bioactive drug delivery systems. The utilization of visible light has not been a conventional practice in PRES. Nonetheless, it has recently garnered substantial interest in creating hydrogels for biomedical applications. A multitude of ongoing studies are currently being conducted to investigate the potential of various materials such as dextran-methacrylate poly(ethylene glycol)-maleic acid [90], hyaluronic acid [91], porcine pericardium (PP) [88], gelatin [92], and diverse elastomers [93,94] in this field. Recently, in situ visible-light crosslinked ESF was prepared by using polyurethane (PU) and light-curable poly(ethylene glycol) diacrylate (PEGDA). The findings showed that the core/shell PU/PEGDA nanofibers were successfully created to limit and control the release of meloxicam [95].

##### Ultraviolet (UV) Radiation

UV radiation (250 nm < λ < 400 nm) has sufficient energy to trigger crosslinking, augmenting electrospun materials’ mechanical properties and stability. This is accomplished through the induction of radical polymerization in the presence of photoinitiators. Numerous studies have investigated the crosslinking of hydrogels, membranes, and ES scaffolds. In this context, the present discussion will focus on in situ crosslinking.

In a study to alter the hydrophobic properties of ionic polyurethane, incorporating D-phenylalanine was deemed a viable modification. Chan et al. [71] fabricated electrospun mats using D-phenylalanine (D-PHI) incorporated with polycarbonate polyurethane (PCNU) and crosslinked using UV radiation during the electrospinning process. The resulting D-PHI/PCNU scaffold exhibited higher hydrophilicity and softness than PCNU films. Upon implantation in Wistar rats, the scaffold degraded slowly over 90 days and maintained its thickness, parallelly demonstrating good merge with the neighboring tissue. Vascular smooth muscle cells (VSCMs) cultured on the scaffold showed excellent growth and proliferation and extended to express α-smooth muscle actin (α-SMA) after 7 days. These findings demonstrate the potential of the D-PHI/PCNU scaffold for tissue engineering applications [96]. In another study, collagen (COL) and polyethylene terephthalate (PET) nanofibers were formulated by PRES using UV irradiation and riboflavin (RIBO) as crosslinkers. Here, 1,1,1,3,3,3-hexafluoro-2-propanol (HFIP) as a solvent effectively fabricated nanofibers of COL, PET, and PETCOL-RIBO. Like COL nanofibers, the PET-COL-RIBO nanofibers had a uniform, porous structure and fiber diameters between 150 and 250 nm. PETCOL-RIBO nanofibers showed advantageous mechanical characteristics after photo-crosslinking compared to COL and PET nanofibers.

Additionally, the relationship between the scaffolds and fibroblast cells was examined, and the findings showed that the cell growth was acceptable. Interestingly, fibroblasts seeded on both COL and PET surfaces showed the usual fibroblast morphology, with a noticeable pattern of thin growths along the length of the nanofibers. However, a low number of cells with a spherical shape were seen on the PETCOL-RIBO surface [97].

More detailed information and guidance related to the most common photopolymerization strategies, various types of photopolymerizable functional groups, photoinitiators, and their compatibility data can be found in the literature [87,98].

##### Incorporation of Functional Group Methyl Acrylate into Polymers

PRES involves a diverse range of polymer modifications before RES. These modifications can be made by adding functional groups such as methylacrylate groups, alkenes, thiols, and nitrene, which promote radical polymerization reactions in the presence of UV radiation.

A portion of the polymerization must be completed beforehand to obtain the ideal viscosity for the spinning process when using functional methacrylates in PRES. For instance, the polymerization of poly(hydroxyethyl)methacrylate (PHEMA) was started with a heat initiator and continued with a photoinitiator through the RE process [99]. Atomic force microscopy imagery of the resulting fibers, which had an average diameter of between 50 and 800 nm in water and showed elastic recovery of the scaffold, supported this claim. It is worth mentioning that one of the initial attempts at photoinduced crosslinking involved modifying poly(methyl methacrylate-co-2-hydroxyethyl acrylate) by adding a cinnamoyl group to enable UV photo-crosslinking of the polymer [99].

In another study, poly(2,3-dihydroxy carbonate) was synthesized by Wu and coworkers, and then a UV-responsive methacrylate group was added. Subsequently, employing different degrees of methacrylation, radiation exposure time, intensity, and use of poly(ethylene oxide) as an agent to boost entanglement, this polymer was electrospuned with on-site/in situ UV crosslinking [100]. The variations in the methacrylation degree impacted the crosslinking degree, which, in turn, impacted several material characterization factors. Successful reaction completion was verified by FT-NIR analysis, which also revealed variation in the methacrylation bands. Morphological analysis showed that fibers, regardless of UV crosslinking, were successfully manufactured, but only the crosslinked scaffolds maintained their structural integrity after being submerged in chloroform for a day. The ESFs also displayed amorphous characteristics after crosslinking, suggesting superior thermal resistance compared to the uncrosslinked fibers, and the fiber diameter climbed with increasing crosslinking degree. It was demonstrated that manipulating the initial synthesis reaction could control the fiber properties. The findings indicated that higher crosslinking levels increased both elastic modulus and tensile strength while reducing the biodegradation rate [100]. The scaffold material exhibited favorable cytocompatibility, as evidenced by the superior attachment and growth of cells on its surface compared to the control. This observation highlights the potential of the scaffold for tissue engineering applications.

Sun et al. aimed to create a vascularized scaffold to improve the survival of skin grafts after plastic surgery [101]. To achieve this, they incorporated methacrylate groups into gelatin, a biopolymer, through a reaction with its amine-containing side chains, making it photo-crosslinkable. They could control the mechanical and biodegradation characteristics of the scaffold by adjusting the amount of crosslinker and methacrylate content. The electrospun scaffolds were capable enough to uphold cell adhesion, in vitro proliferation, and translocation and were found to rapidly form 3D vascular networks in vivo. Moreover, the scaffold’s fibrous nanostructure and hydrogel softness were maintained, making it an appropriate scaffold for tissue engineering applications.

In research by Ferreira and colleagues, Irgacure^®^ 2959 was used as the photoinitiator to prepare a composite of polycaprolactone (PCL) and photo-crosslinkable methacrylate gelatin (GelMa) that was then photo-crosslinked under UV light. The findings demonstrated that each substance had a comparable morphology and was biodegradable, with the rate of degradation being influenced by the amount of gelatin used. Gelatin-infused fibers showed decreased water contact angles and improved biological qualities. Blood compatibility tests revealed negligible thrombogenicity—as low as 10% for mixes containing more gelatin—and no erythrocyte membrane disruption. The presence of the materials had no impact on how Normal Human Dermal Fibroblast (NHDF) cells attached and grew, and they showed no morphological variations. These results demonstrate the flexibility and benefits of using PRES to create 3D constructs for tissue engineering. They also suggest that the manufactured materials have favorable properties, which makes them a possible choice for use as vascular grafts [102].

##### Thiol-ene Polymerization

**Figure 6:**
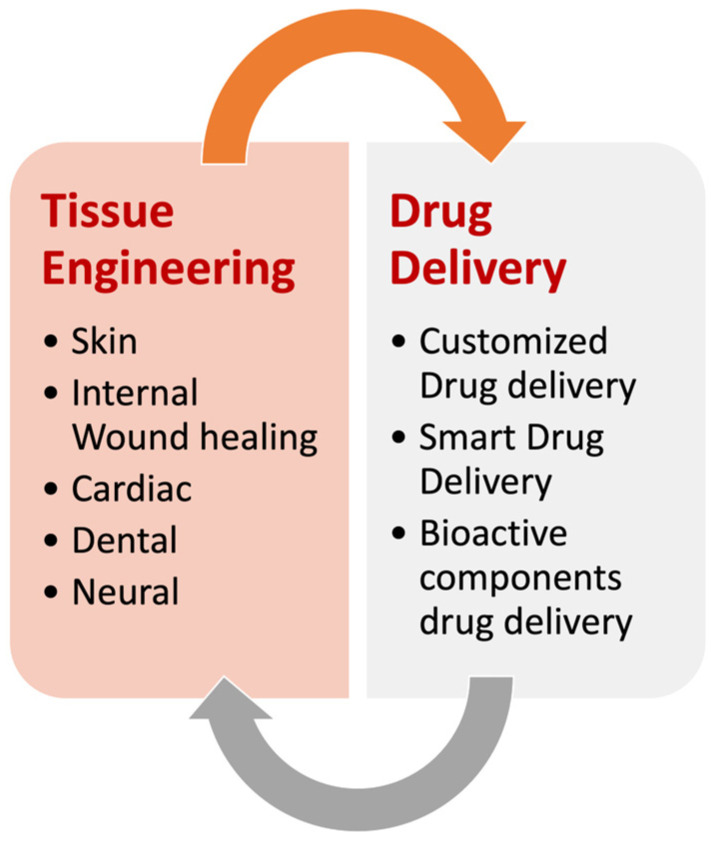
Biomedical application of RES.

The range of polymer structures that can be created by combining various functional groups is significantly increased by the ability of polymers with terminal or internal alkenes to engage in photo-crosslinking with thiols (R−SH) to form a thioether. It is worth mentioning that the reactivity of these alkenes changes depending on the kind and degree of double-bond substitution [103]. A recent study describes a novel technique for producing ultrathin rubber fibers sustainably and efficiently without using solvents, crosslinkers, or photoinitiators. The process involves using liquid maleinized polybutadiene (PB) polymers, which enables rapid curing during electrospinning through radical abstraction and photoinduced ring-opening reactions. Pure liquid maleinized PB polymers alone were inadequate to produce the desired fiber morphology. Hence, the photoinitiator trimethylbenzoyl diphenylphosphine oxide (TPO) and the multifunctional thiol-based crosslinker trimethylolpropane tris(3-mercapto propionate) were added. Thiol crosslinking, esterification of maleic anhydride moieties, and oxidation of polybutadiene chains were all parts of the following the photoinduced crosslinking process. With the help of the better formulation, crosslinked rubber fibrous membranes measuring 48 m in diameter were produced. These membranes had high insolubility (>80%), good thermal characteristics, a low Tg, and a distinct hydrophobic and oleophilic character. These outcomes highlight the potency of the enhanced formulation and its promise for various uses (Figure 6). The method converts liquid low-molecular-weight polybutadienes into crosslinked rubber fibers in a single step without using solvents or heat [104].

In another study, 2,2 dimethoxy-2-phenyl acetophenone was used as a photoinitiator along with a dithiol crosslinker to electrospin unsaturated aliphatic polyglobalide (PGl) into established fibers with an average diameter of 9 μm. By incorporating these additives during the spinning process, the fibers were able to undergo in situ crosslinking, ultimately forming an amorphous material. Remarkably, this material maintained its fibrous morphology even after swelling up to 14% in tetrahydrofuran (THF). The study showcased the potential of PGl fibers as a scaffold for cell growth by illustrating that mesenchymal stem cells (MSCs) implanted on both crosslinked and non-crosslinked fibers exhibited remarkable biocompatibility and substantial proliferation. The research also showed direct hydrophobic molecule loading onto the crosslinked PGl fibers, including rhodamine B and the antialiphatic polyester inflammatory medication indomethacin. This method offers encouraging prospects for developing drug-loaded polyester frameworks for biomedical applications and TE purposes, as it demonstrated enhancements over the commonly used aliphatic polyesters [105].

In another study, a dual-syringe setup contained two separate aqueous solutions. The first injection was composed of hyaluronic acid with thiol functionality and PEO, while the second contained poly(ethylene glycol) diacrylate (PEGDA) [106]. Initial results from RE showed a homogeneous spread scaffold with an average diameter of 50–300 nm. Notably, the crosslinking reaction between the thiol-functionalized HA and PEGDA occurred at room temperature in less than 10 min without UV radiation. Fibroblasts were able to penetrate the scaffold thanks to the ensuing fiber network and create 3D dendritic networks.

##### Nitrene Formation

In addition to the alkyne–azide cycloaddition reaction, phenyl-azide groups can undergo photoinduced disproportionation, forming a highly reactive nitrene intermediate. This procedure can encourage reactions of addition to an alkene or produce an intermediary dehydroazepine that can interact with primary amines.

A new technique for crosslinking polylactic acid (PLA) was recently proposed by researchers, wherein a polyfunctional azido compound is used as a crosslinker for photocuring of a polylactic acid pluronic copolymer, which does not possess any alkene or alkyne groups [107]. This approach is built upon a previously established method for UV-induced crosslinking of polyesters [108], which leverages the UV-induced aryl azide group to create highly reactive nitrene species capable of integrating with the carbon–hydrogen bonds of the polymer’s backbone, thereby enabling crosslinking through amine groups. The researchers used a straightforward but elegant method to crosslink various non-prefunctionalized polymers using a polymeric multiazide crosslinker. Degradable elastomers for soft tissue engineering were also created using this method.

In research by Lin and Tsai, polyacrylic acid chains (PAA) were functionalized with azido groups, which were combined with gelatin before electrospinning to photo-crosslink gelatin fibers [109]. The azido crosslinked gelatin and the traditional glutaraldehyde (GTA) crosslinked gelatin were examined for their mechanical properties, cell attachment, and cytotoxicity. Due to the fabrication of a crosslinked lamina on the surface that prevented GTA vapor from diffusing into the interior of the fibers, their DSC findings showed that the crosslinking achieved by GTA was observed to be nonuniform across the fibers. The cellular compatibility of PAA-AZ crosslinked fibers was better, and no cytotoxicity was found. The authors added hydroxyapatite nanoparticles to the fibers to increase the gelatin ESFs’ bioactivity, which led to better cell mineralization and verified the fibers’ suitability for tissue engineering applications [109].

##### Gamma Radiation

In many instances, it requires more energy to reach the desired crosslinking extent necessary for preserving the fibrous structure. Also, creating a fibrous matrix through UV-light photo-crosslinking can occasionally be challenging. Therefore, utilizing high-energy gamma radiation for photo-crosslinking might offer the best alternative. Dargaville et al. investigated the electrospinning procedure of the low-molecular-weight poly(trimethylene carbonate-l-lactide) by acrylating the polymer’s end groups [110]. Because the polymer’s lower glass transition temperature and slow crosslinking kinetics caused fiber fusion, their efforts to crosslink the fibers in situ or post-electrospinning using UV light were unsuccessful. Additionally, the UV lamp’s heat hastened the fusion of the fibers. For a polymer solution to form strands that can withstand the voltage’s stretching without disintegrating into droplets, the concentration must be higher than the critical entanglement concentration. This was not possible, though, because of the low MW copolymer used.

Gamma irradiation was used to create steady fiber morphology. Despite the possibility of causing deterioration, gamma radiation had several advantages, such as excellent effectiveness, sterilization impacts, and elimination of potentially toxic photoinitiators. In sequential mechanical and fatigue testing, the resulting fibers demonstrated excellent resilience and elasticity, essential for applications in mechanically changing conditions like the vascular system. The moduli also fell into the region of human arteries. Additionally, the scaffolds improved human mesenchymal stem cell development and proliferation, making them viable options for vascular tissue engineering [110]. Gamma ray-induced chain degradation can potentially compromise the mechanical properties of polymers, even though gamma irradiation is commonly employed to induce crosslinking and improve polymer strength. For example, polyamide 66 (PA66) fibers can experience adverse effects on their properties due to irradiation [111]. Through irradiation-induced crosslinking, however, the addition of triallyl cyanurate (TAC) at a low level was able to fix this problem and enhance the mechanical properties of the PA66-TAC fibers. Gamma radiation is a sanctioned technique for achieving sterilization methods in various biomedical applications, and its impact on cell response is crucial. Gamma radiation impacts the molecular weight, crystallinity, and mechanical characteristics of polycaprolactone (PCL) fibers in this instance. However, comparing ethanol immersion and gamma irradiation sterilization showed that both equally supported cell proliferation [112].

## 3. Biomedical Application of RE

RE enables the production of nanofibers with customizable properties through varied crosslinking strategies. The customized characteristics of the resulting scaffolds render them suitable for a wide range of biomedical applications. RE is particularly advantageous in the biomedical arena because of its ability to fabricate crosslinked hydrogel nanofibers that can serve as scaffolds for tissue regeneration. This method allows for concurrent crosslinking during the electrospinning process, fine-tuning of crosslinking density for specific tissue engineering needs, preservation of the nanofibrous structure after hydration, and potential use in wound healing and tissue regeneration (Figure 6).

### 3.1. Advantages of RES for Biomedical Applications

Continuous attempts to produce the ideal biomimetic nanofibrous scaffolds that resemble extracellular matrices have directed researchers to utilize RES to fabricate scaffolds with optimal fibrous orientation (anisotropy), porosity, conductivity, and mechanical properties [113]. Reactive electrospun nanofibers offer many benefits over conventionally prepared ESFs. Such benefits include enhanced mechanical properties, stability and durability, diverse fiber architecture, and versatility in functionality and controlling the release of loaded drugs.

#### 3.1.1. Enhancement of Mechanical Properties

In many reported studies, RES was utilized with various crosslinking approaches to enhance the mechanical properties, biodegradation, and stability of the ESFs fabricated using water-soluble polymers [114,115]. Crosslinking is typically classified into physical and chemical crosslinking. Hydrogen bonding, electrostatic interaction between ions, and crystallization acting as binding points between molecules are the main factors affecting this type of crosslinking. Chemical or photo-crosslinking, on the other hand, results when covalent bonds are formed between the molecular chains of a polymer, thus increasing its molecular weight and improving mechanical properties such as strength, stiffness, abrasion resistance, hardness, thermal stability, etc. The kinetics of crosslinking reactions are governed by the chemical structure of the reactants and crosslinkable functional groups involved and their concentration. Therefore, crosslink density and reaction rate can be controlled by varying the concentration of crosslinker and reaction conditions [87,94]. The higher the crosslinking density, the more mechanical strength the fabricated fibers attain and the lower their degradation rate.

Reported investigations showed that in situ UV crosslinking of various proportions of polyethylene glycol methacrylate in polyurethane-based scaffolds improved mechanical properties, specifically increased tensile strength. The scaffolds with higher percentages of the methacrylate moiety displayed greater tensile strength than those with less methacrylate and more polyurethane. Additionally, the scaffolds exhibited enhanced hydrophilicity, as evidenced by increased water uptake and changes in contact angle measurements [116,117]. The scaffolds’ cytocompatibility with Human Umbilical Vein Endothelial (HUVE) cells was examined and showed nontoxic behavior [116].

Another investigation reported a nanofibrous polymer of methacrylated gelatin (GelMA) and dopamine (DA) nanofibers. By altering the methacrylates substitution levels of gelatine, the nanofibrous hydrogels displayed controllable adhesive and mechanical characteristics. In comparison to gelatin nanofibrous hydrogels, the optimized GelMA60-DA displayed 2.0 times greater tensile strength (2.4 Mpa) with an approximate 200% elongation, 2.3 times higher adhesive intensity (9.1 kPa) on porcine skin, and 3.1 times higher water vapor transfer rate (10.9 kg m^−2^ d^−1^). Parallel to this, the GelMA60-DA nanofibrous hydrogels promoted cell development and quickened the healing of wounds [118].

In another similar but recent study, researchers blended gelatin with a methacrylate dextran derivative, which was subsequently photo-crosslinked to produce fibers with a diameter ranging from 0.30 μm to 1 μm. The blend exhibited significantly enhanced mechanical properties, as evidenced by a Young’s modulus of 40 Mpa. Furthermore, the blend demonstrated an impressive water sorption capacity of 1500–2000% within 20 min. Given that cells could adhere and proliferate on the material, it was concluded that these fibers represent a hopeful contender for soft tissue engineering purposes owing to their combined mechanical strength and favorable biocompatibility [119].

#### 3.1.2. Enhanced Stability and Durability

Natural polymers have generally outperformed synthetic polymers regarding TE capabilities because of their excellent biocompatibility, favorable host immune reaction that promotes tissue remodeling, and capacity to create an instructive microenvironment for tissue remodeling. However, these natural polymers exhibit weak stability and durability in aqueous media. In contrast, hydrophilic characteristics are expected in synthetic polymers appropriate for biomedical uses. Although this is the case, synthetic polymers can still be unstable in aqueous environments. Thus, by adding crosslinking agents to the fibers using various crosslinking techniques, as previously stated, it is possible to increase the stability and durability of electrospun nanofibers.

Xu et al. studied the use of methacrylated linear polyethyleneimine (M-PEI) to produce scaffolds through PRES intended for tissue engineering purposes [120]. The researchers tried in situ crosslinking using UV light and electrospun different concentrations of methacrylated M-PEI in ethanol. The study’s findings demonstrated that the hydrophilic properties of the polymer substance and the PEI’s low molecular weight posed obstacles to the development of fibrous structures. This caused the chain entanglement to decline below the essential critical threshold for fiber production even at concentrations ranging from 10 to 30% *w*/*v* and methacrylation degrees between 3% and 59.2%. The researchers used high-molecular-weight PVP at 2% *w*/*v* as an entanglement booster to get around this problem. SEM showed that porous fibrous structures were successfully formed at 10%, 20%, and 30% PEI ratios. The scaffold was additionally made impervious to ethanol, water, and culture medium thanks to UV crosslinking, maintaining the porous structure. Mechanical testing showed that the visible tensile strength increased as the crosslinking degree increased. Although the study’s acrylation degrees were only up to 14.8%, the fabricated scaffold’s use for TE applications may be enhanced by conducting additional tests on its fiber properties regarding pore size, porosity, and in vitro cell toxicity [120].

In another study, Theron, J.P. et al. modified commercial medical polyurethane using acyl chlorides [121]. After that, the altered polyurethane was electrospun and in situ crosslinked with UV light to create vascular grafts with the required properties. SEM images successfully verified the electrospun polyurethane, and after UV crosslinking, the polyurethane showed increased resistance to H_2_O_2_ and AgNO_3_. Burst pressure tests revealed that the created grafts had the necessary characteristics, though compliance was decreased because of the graft’s comparatively low porosity. The changed polyurethane also had less hysteresis and creep. Further research is needed to examine the mechanical characteristics of crosslinked and uncrosslinked electrospun grafts with various degrees of modification and assess the mutated polyurethane’s cytotoxicity. Despite this, the development of low-toxic and effective UV crosslinking and the use of chemical alteration to regulate the rate of crosslinking has shown the potential to extend the range of applications for medical polyurethane. The study demonstrated the effectiveness of this method for crosslinking pullulan in electrospinning applications, as discussed later in this review article. Stable pullulan nanofibers were successfully produced using an in situ crosslinking electrospinning technique with glutaraldehyde and sulfuric acid, improving thermal stability and significantly increasing water absorption rates [122].

#### 3.1.3. Better Control of ESF Architecture and Drug-Release Rate

As discussed earlier, the functionalization and crosslinking of ESFs result in optimum porosity, low matrix degradation, and controllable drug release due to the formation of three-dimensional networks. The changes in the crosslinking density and, consequently, the mechanical strength and crystallinity of the fabricated ESFs would tremendously impact the release rate of drugs from the fabricated ESFs, including the extent of burst and lag effects [123]. Crosslinking was also reported to affect the water-binding ability of the fabricated polymeric scaffolds due to their hydrophilic nature and the three-dimensional structure formation, which was demonstrated to impact drug release and the architecture of the crosslinked matrices [124,125].

The development of biomaterials with core–shell structures has emerged as a promising approach to achieve controlled release of susceptible biomolecules while also enabling the incorporation of natural biomaterials as shell layers to impart biofunctionality to surfaces. Fabrication of coaxial electrospun nanofibers loaded with various concentrations of two distinct proteins, BSA and EGF, was reported. The two proteins were integrated into the pure GT shell and hybrid GT/PCL shell at various weight ratios by adjusting the coaxial electrospinning parameters. FM and TEM imaging verified the production of core–shell nanofibers with consistent protein distribution. A decline in fiber diameter, a rise in Young’s modulus, and a significant reduction in ultimate strain were all caused by increasing the GT proportion in the shell. In comparison to blend scaffolds, coaxial scaffolds showed more sustained-release patterns. Protein release and burst release were both accelerated by higher GT concentration in the shell material. In addition to enhancing mechanical performance, the crosslinking treatment regulated the intense burst and total release. The greatest modulus and final tensile stress were seen in the GT7P3-crosslinked scaffold, which was correlated with elevated cell spreading and proliferation. The release of 0.05% BSA and EGF showed a minor burst release, followed by a gradual release rate, demonstrating the scaffold’s potential for other types of proteins. These scaffolds might make suitable choices for TE applications, according to the encouraging in vitro cell culture findings. Thus, the study demonstrated that mechanical characteristics and release rates can be tuned to suit particular needs by varying the mix proportion of both synthetic and natural polymers as the shell layer and using crosslinking in the coaxial system [126].

A new technique was developed to reduce the toxicity of cyanoacrylates, which are used to close wounds. This method makes use of n-octyl-2-cyanoacrylate, which is certified by the FDA and approved as medicinal glue, and an airflow-assisted electrospinning procedure known as “in situ precision ES”. An airborne anionic initiator starts the in situ crosslinking process, forming a thin fibrous barrier on the wound surface. Additionally, the cultured liver tissues’ amino groups support crosslinking, which results in full wound closure and the halt of additional blood loss. This technique reduces the required dose by about 80% compared to traditional spraying, reducing the harmful effects of cyanoacrylates. Histological studies of the hepatocytes performed seven days later proved the effectiveness of this method. Looking deeper into the study, one can conclude that more information on the machine configuration, chemical and morphological characterization, and electrospinning process parameters would be helpful. In addition, the researchers reported using n-octyl-2-CA on liver cells although it was FDA-approved for external use only. However, using n-butyl-2-CA, which was approved for internal use, would have been a better option. Moreover, the sample size varied in their experiments, and only rat liver was used as an example of the application [127].

### 3.2. Tissue Engineering (TE) Applications

In TE, fabricating electrospun scaffolds that precisely replicate the ECM behavior is paramount. Each cell type demands a bespoke scaffold possessing properties that can be fine-tuned to meet specific requirements. These properties, comprising porosity, mechanical characteristics, and scaffold response in a biological milieu, are contingent on the cell type. RE is an auspicious technique that can craft scaffolds with customizable attributes tailored to exact TE needs. In situ crosslinked electrospun scaffolds find application in several domains, such as skin tissue engineering for wound healing, cardiac TE, dental TE, and nerve TE.

#### 3.2.1. Skin TE Applications

The TE for wound-healing applications and skin regeneration holds interest for the creation of tissue-engineered biodegradable artificial tissue substitutes with ECM-imitating properties that control the relationship between the material and the living environment. Double-layer mats composed of fish collagen (FC) and PCL were reported, which are covalently bonded by chitooligosaccharides (COS) via carbodiimide chemistry. The scaffold showed better hydrophilicity, swelling, and mechanical integrity. Following crosslinking, the FC content was associated with a shift in fiber diameter. Effective fibroblast and keratinocyte cell adhesion, infiltration, and proliferation were seen in in vitro experiments. The bilayered nanofibrous scaffold accelerated dermal tissue maturation, reepithelialization, and curing when placed on a penetrating wound in a rodent model [128].

Another novel approach used elastin-like recombinamers involving in situ mixing of two “clickable” elastin-like recombinamers (ELRs) during electrospinning without crosslinking agents. The culture of keratinocytes and fibroblasts on ELR-click fibers showed cytocompatibility, as evidenced by adhesion, proliferation, fluorescence, immunostaining, and histology studies [129]. Crosslinked electrospun mesh containing antibacterial components was also reported to have potential applications in wound healing. Villeges et al. conducted a study in which chitosan (CS)/polyethylene oxide (PEO) nanofibers (NFs) were prepared with zinc oxide (ZnO_2_) nanoparticles and UV-crosslinked using a pentaerythritol triacrylate (PETA) photoinitiator. The CS/PEO/ZnO fibers that were crosslinked for 100 min exhibited the highest swelling capacity in aqueous solution, with a swelling rate of 770%. Furthermore, UV-crosslinked CS/PEO/ZnO NFs showed antibacterial properties against several bacterial strains, including *S. aureus*, *E. coli*, *S. epidermidis*, and *P. aeruginosa* [130].

Photoinitiated in situ crosslinking of polyvinylpyrrolidone (PVP) with a benzophenone molecule, a UV-sensitive, with-egg lysozyme (LY), was effectively incorporated into fibrous mats. The findings demonstrated that many disrupted bacteria were seen in AFM images when S. aureus was cultured with pure LY and PVP-BP-LY, suggesting lysozyme hydrolysis of the cell walls’ peptidoglycan. Additionally, compared to pure BP, the PVP-BP covering was less harmful to fibroblasts, indicating that adding BP molecules to PVP fibers lessens the cytotoxicity of pure BPs [131].

In a separate study, silver nanoparticles were incorporated into a polyvinyl alcohol (PVA) matrix in situ crosslinked using GT. The PVA strands’ hydroxyl groups functioned as the AgNPs’ reactive sites and stabilizers. Glutaraldehyde concentration can be adjusted to adjust the crosslinking degree and produce partly or completely crosslinked PVA nanofibers with embedded AgNPs. The extent and rate at which silver ions were released into the surrounding aqueous solution depended critically on the degree of crosslinking. Due to the creation of acetal groups during crosslinking, it was found that this aqueous solution is pH-responsive and acid-labile. Both partially and fully crosslinked PVA nanofibers doped with AgNPs demonstrated excellent antibacterial properties against S. aureus with minimal cytotoxicity. The controlled-release technique used in the research has a lot of promise for creating pH-responsive, environmentally friendly materials for wound-healing applications [132].

#### 3.2.2. Internal Abdominal Wound Healing

The use of a composite bilayer wrap with selective bioactivity is a novel method to decrease intra-abdominal adhesions and improve anastomotic healing following intestinal surgeries. RE was used to create a crosslinked gelatin mesh in a double-barrel syringe with isocyanate as the crosslinker. A PEG foam coating was applied to the bioactive mesh to avoid intra-abdominal adhesions. According to preliminary tests, the adhesive composite bilayer wrap retained a maximum shear strength greater than fibrin glue and comparable to the marketed adhesion barrier. The hydrogel foam samples were still extant on day 21, while the gelatin meshes had lost their mechanical integrity by day 7. These findings allowed for an early assessment of adhesion prevention because they showed that the deterioration profiles of every element were within the desired range. However, a lengthier study is necessary to fully assess the hydrogel foam’s in vivo degradation rate. The wrap initially showed promise in avoiding surgical adhesions, and it was determined that it could do so while also reducing adhesions and enhancing anastomotic healing. Creating a therapeutic strategy that simultaneously deals with anastomotic leakage and intra-abdominal adhesions can significantly enhance patient results and lessen the need for extended hospital stays and additional surgeries [133].

#### 3.2.3. Cardiac TE Applications

**Figure 7:**
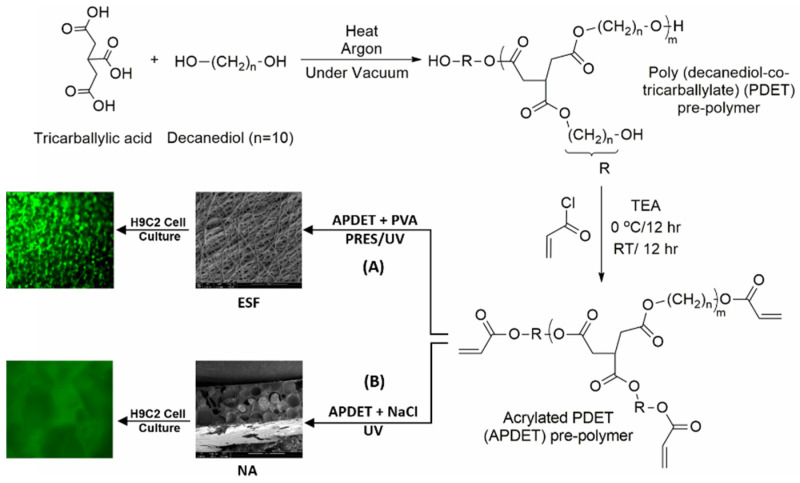
Schematic illustration of the PDET synthesis, acrylation, and further crosslinking to prepare (**A**) electrospun fibers (ESFs) using UV-based photoreactive electrospinning (PRES) or (**B**) UV photo-crosslinked scaffolds using the NaCl particulate leaching technique (NA). (Reproduced from reference [134] with permission).

The scaffold intended for cardiac tissue engineering must possess suitable mechanical properties, such as an elastic character supporting cardiac functionality, including contraction and relaxation. Additionally, scaffold pore size is essential in promoting cell proliferation and growth. Ismail et al. studied the in situ UV photo-crosslinked electrospun nanofibers using poly (1–10, decandiol-co-tricarbllyate) and found that the prepared scaffold held a porosity of approximately 70% (Figure 7) [134]. The scaffold’s mechanical behavior demonstrated an elastomeric nature that could withstand cardiac functions such as relaxation and contraction. Ultimately, the scaffold exhibited higher cell biocompatibility and robust growth and proliferation of myocardiocytes. These results indicated that this electrospun scaffold is an ideal candidate for cardiac tissue engineering applications.

#### 3.2.4. Dental TE Applications

**Figure 8:**
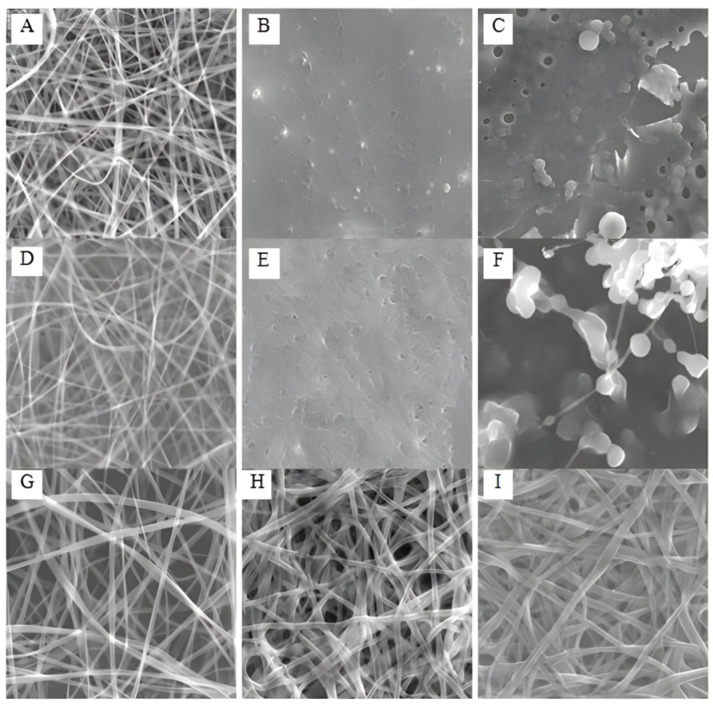
SEM of electrospun Zein nanofibers. Native Zein: (**A**) as electrospun, (**B**) after water immersion, (**C**) after 75% ethanol immersion. Methacrylic Zein: (**D**) as electrospun (298 ± 102 nm), (**E**) after water immersion, (**F**) after 75% ethanol immersion. UV-crosslinked methacrylic Zein (348 ± 146 nm): (**G**) as electrospun, (**H**) after water immersion, and (**I**) after 75% ethanol immersion (336 ± 152 nm) (all magnification: ×5k) (reproduced from reference [136] with permission).

The area of periodontal TE requires suitable frameworks that possess favorable mechanical properties and antibacterial characteristics. A recent study examined nonwoven scaffolds comprising 16% gelatin and 5% hydroxyapatite. Water-soluble polyethylene glycol was added to the scaffold to create a porous structure and crosslinked by heating the fiber mesh to create a porous structure. The nonwoven scaffolds were successfully populated by human mesenchymal stem cells and periodontal ligament fibroblasts, with greater cell density observed in scaffolds with additional porosity. Metabolic activity was found to be higher in cocultures of both types of cells [135]. In another study, a UV lamp was used during electrospinning in recent research to crosslink Zein, which had been methacrylated to become a photoreactive monomer (Figure 8) [136]. The ESF developed a potent antimicrobial property by adding an antibacterial methacrylate monomer and crosslinking throughout the electrospinning process. Zein can potentially be used in dental and biomedical applications due to the novel method used in the research and the embedded antimicrobial properties.

#### 3.2.5. Neural TE Applications

In neural TE, the scaffold should be able to foster the growth and differentiation of neural cells without causing an adverse reaction or immune response. In recent years, applying mechanical stimuli in addition to biological, biophysical, and biochemical stimuli for spinal cord regeneration has gained attention. A photo-crosslinked gelatin methacryloyl (GelMA) scaffold for mechanical stimulus in spinal cord function regeneration was studied by Chen et al. using RE. Because of the scaffold’s low Young’s elasticity and high stretchability, neuronal cells had a high viable rate and advantageous metabolic environments. In addition, hippocampal neuron cells cultured on GelMA scaffolds displayed longer axes than those in the control group, suggesting the ability of GelMA scaffolds to encourage cell development. GelMA scaffolds were shown to be able to aid the migration and long-term survival of neural stem cells (NSCs) by immunofluorescence staining of Tuj-1-labeled neuron cells that had undergone differentiation from NSCs. According to these findings, GelMA scaffolds may be used as a mechanical stimulus scaffold for spinal cord regeneration [137].

Table 2 summarizes a list of recently reported research on utilizing CRES, PRES, and other RE techniques for TE applications with details on the polymers used, the RES technique utilized, the type of crosslinker or the photoinitiator used, and the photo radiation source.

### 3.3. Drug Delivery Applications

CRES and PRES are both highly capable of producing ESFs customized for drug delivery applications. Their unique properties, which include high surface-area-to-volume ratio, porosity, and crosslinking capabilities, make them very well- suited for tailoring controlled-release smart drug delivery systems and incorporating bioactive ingredients through applying both in situ and post-crosslinking strategies as promising approaches to novel treatments for various diseases and conditions [149].

#### 3.3.1. Customized Drug Delivery Rate and Extent

It was reported that the degree of crosslinking in fabricated ESF and the rate and extent of drug release are directly related [128]. Scientists may fine-tune the drug’s release rate by fiddling with crosslinking and creating highly customized treatments tailored to specific patients’ needs. The CRES technique has demonstrated its efficacy as a cutting-edge drug delivery system by successfully facilitating the sustained release of buprenorphine (Bup) within a polyvinyl alcohol (PVA) and polyvinylpyrrolidone (PVP) matrix. According to a study by Rahmani et al., a nanofiber mat was created for transdermal drug administration that contained Bup-loaded PVP and Bup-loaded PVA/PVP. According to the FT-IR findings, the electrospinning did not compromise the drug’s chemical integrity. Compared to Bup/PVP, Bup/PVP/PVA nanofibers showed superior physical and chemical characteristics. The length of the drug-release period increased due to the crosslinking of nanofibers. [129]. Simultaneously, polyvinyl alcohol/chitosan nanofibers were loaded with gentamicin sulphate. Increased crosslinking density displayed outstanding antibacterial capabilities due to the controlled-drug-release capabilities, making them ideal for advanced wound dressings and tissue engineering applications [130]. In another research study, the modified release of dexamethasone (DMS) was studied using PLLA and gelatin, which were crosslinked with GTA to stabilize the structure. Crosslinking intensity has considerably changed the in vitro release of integrated DMS, which is essential for biological applications such as tumor therapy. Including gelatin affected fiber diameter and surface hydrophilicity [131].

PRES, on the other hand, exhibited precise control over the release of meloxicam, demonstrating its efficacy as an advanced drug delivery technique. A study that used polyurethane, polyethylene glycol (PU), and light-curable poly(ethylene glycol) diacrylate (PEGDA) as drug carriers was recently reported. The fabricated nanofibers were created in monolithic, blended, and core/shell configurations that were exposed to visible-light-photocured in situ crosslinking. The findings showed that the core/shell PU/PEGDA nanofibers were successfully created to sustain and control the release of meloxicam. This approach offered a viable one-step way for constructing nanofiber-based drug delivery systems without dissolving or harming the nanofibers during crosslinking [90].

Many other recent studies investigated PRES in situ crosslinking of microspheres loaded with the drug into ESFs. Electrospinning of the microsphere suspension in conjunction with pulsed voltage to produce modified electrospun polyvinylpyrrolidone (PVP) fibers integrating drug-loaded polycaprolactone (PCL) or polyethersulfone (PES) microspheres was studied. This technique improved mechanical strength, optimized drug-release profiles, increased the system’s ability to carry more drugs, and eliminated burst effects. The modified electrospun mats underwent UV crosslinking to produce flexible ethanol- and water-insoluble fibers, which impacted the degradation rate, mechanical strength, and transport characteristics. These UV-crosslinked PVP-based mats containing PCL or PES microspheres demonstrated promise as highly adjustable drug delivery systems, since this method’s adaptability allows for various alterations [132].

In another recent study, thermal crosslinking was carried out in PVA. The researchers created graded membranes with controlled crosslinking by including graphene nanoplatelets (GNP) and chlorhexidine (CHX) in the formulations. GNP was added, which improved thermal conductivity, avoided delamination problems, and increased mechanical strength. These graded membranes demonstrated the potential for customized drug delivery systems, demonstrating a multimodal drug-release pattern by releasing CHX quickly at first and maintaining consistent release over time [133].

Electrospinning was also combined with post-thermal treatment to create stable nanofibrous substrates incorporating diclofenac from an aqueous solution made of polyethylene glycol (PEG) and carboxymethyl cellulose (CMC). A two-step, needleless electrospinning method was used to produce bicomponent-blend nanofibers with varying crosslinking densities by adjusting the concentrations of the butane tetracarboxylic acid (BTCA), green polycarboxylic crosslinker, and the catalyst, sodium hypophosphite (SHP), as well as the temperature. The kinetics of diclofenac release and biocompatibility with human skin fibroblast cells were used to assess the characteristics of crosslinked nanofibers. The total area of nanofibers increased in thermally crosslinked samples while the fiber diameter, pore volume, and pore size dropped. Swelling and quartz crystal microbalance experiments revealed that as BTCA concentrations increased, the crosslinked mats became more stable and capable of swelling. All crosslinked mats had identical discharge kinetics, although a brief burst accompanied by a diffusion-controlled release of diclofenac was observed. Additionally, the drug release followed a non-Fickian diffusion process. The study showed that although the polymers, crosslinker, and high quantity of drug did not promote the proliferation of fibroblast cells, they did not decrease their viability either [150].

#### 3.3.2. Smart Drug Delivery System

In recent years, significant strides have been made in the field of drug delivery, with a focus on developing intelligent and self-regulated systems to enhance therapeutic efficacy while minimizing adverse effects [151]. CRES of gelatin and thermosensitive polymer poly(N-isopropyl acrylamide) (PNIPAM) was utilized to fabricate a delivery system for the anticancer drug doxorubicin hydrochloride, which is capable of controlling its release in response to the surrounding environmental temperature as the electrospun scaffolds swell and deswell [152].

PRES was utilized to design intelligent drug delivery systems. Recent research successfully developed a coaxial electrospun nanoplatform for self-regulated drug delivery based on photo-crosslinked polymer poly(N-isopropylacrylamide-co-N-isopropylmethacrylamide) (poly(NIPAAm-co-NIPMAAm)) hydrogel with biomimetic structural properties. Using a thermoresponsive hydrogel P(NIPAAm-co-NIPMAAm) in the core loaded with a rhodamine B (RhB) as a drug model and a poly-l-lactide-co-caprolactone (PLCL) shell that served as both a protective layer and a reservoir during drug release, this system showed self-programmable release rates. When exposed to temperatures above the body temperature, the hydrogel core underwent reversible physical changes crucial to the regulation process. The system showed temperature-dependent drug delivery kinetics driven by temperature-controlled desorption [153].

#### 3.3.3. Therapeutic Protein-Loaded Scaffolds

The concept of in situ crosslinking was innovatively introduced to achieve the sustained release of bioactive ingredients from photoreactive acrylates crosslinked electrospun matrices. In this approach, at least one polymer was gelatin, while the other was a natural or synthetic dendrimer. The approach marked an advancement in the controlled release of bioactive compounds, demonstrating the potential of combining different polymer matrices for electrospun-based drug delivery applications [154].

In recent research on fabricating gelatin-based ESFs, PRES and CRES were simultaneously utilized with methacrylated gelatin and diisocyanate. It was reported that the model protein drugs fluorescein isothiocyanate, labeled bovine serum albumin (FITC-BSA), and tetramethylrhodamine isothiocyanate, labeled bovine serum albumin (TRITC-BSA), were rapidly released from the PRES-prepared gelatin–methacrylate system, with 48 ± 12% released on day 1 and 96 ± 3% released on day 10. On the other hand, the protein in the CRES crosslinking system was conjugated to the gelatin via the diisocyanate. It required the degradation of the gelatin before diffusion out of the fibers. The CRES crosslinking system showed a more long-lasting release profile, with only 7 ± 5% released on day 1 and 33 ± 2% released on day 10. A subsequent release analysis of a cospun mesh with two separate crosslinked fiber populations confirmed the ejection of multiple growth factors with distinct release kinetics from a single mesh [155].

Isorhamnetin glycoside (IRG), which is a natural phytochemical that is present in the species Opuntia ficus-indica (OFI), was incorporated with the gelatin (GL) scaffold for wound-healing application, and it was shown that incorporating bioactive proteins into the scaffold is difficult due to protein denaturation in various environmental conditions. Two distinct concentrations of GL and OFI flour were evaluated to produce nanofibers. The possibility of fabricating and characterizing IRG-loaded gelatin (GL) force-spun fibers crosslinked with glutaraldehyde (GTA) was investigated. The GL concentration substantially impacted the IRG release, and the fibers had nanoscale diameters. After 72 h, the GL/OFI2 nanofiber had a total IRG release of 63%. Both nanofibers were suitable for human fibroblast and skin cells, and GL/OFI1 nanofibers showed characteristics that made them useful as drug-release devices. In the research, a novel bioinspired GL-based drug delivery system is demonstrated. It may be used to deliver controlled amounts of phytochemical medications for tissue regeneration and the treatment of wounds [156].

Table 3 summarizes a list of recently reported research on utilizing CRES, PRES, and other RE techniques for drug delivery applications with details on the polymers used, the RES technique utilized, the type of crosslinker or the photoinitiator used, and the photo radiation source.

## 4. Biocompatibility Testing of Crosslinkers and RES-Fabricated Fibers

### 4.1. In Vitro Cell Viability, Cytotoxicity, and Cell Proliferation

Cell viability, cell proliferation, and cytotoxicity tests using cultured cells are the most used in vitro assays for assessing cytocompatibility and the impact of using various polymers, solvents, and crosslinking agents on cell viability and the fabricated ESF cytotoxicity (the toxic quality of the prepared fibers on the cells) [162]. The measure of the number of living cells compared to the control alongside cytotoxicity tests helps us to understand how cells’ health status and counts reflect ESF toxicities.

The use of in vitro cytotoxicity and cell viability assays has some advantages when they are used for the cytocompatibility assessments of fabricated ESF, such as speed, reduced cost, and potential for automation and tests using human cells, which may be more relevant than some in vivo animal tests. However, they have some disadvantages because they are not yet technically advanced enough to replace in vivo testing [163]. The recent advances in artificial intelligence (AI) and machine learning (ML) in toxicological studies tremendously improved toxicity predictions. They provided more accurate and efficient methods for identifying the potentially toxic effects of tested materials before they are tested in human clinical trials [164]. For details on the different techniques of in vitro cell viability, cytotoxicity assays, and cytocompatibility of various crosslinking agents and photoinitiators, we recommend the reader refer to the following references [165,166].

In a study, the in situ photo-crosslinked fibers from methacrylated cellulose acetate butyrate (CABIEM) and collagen-modified versions of those fibers were fabricated. The ECV304 and 3T3 cells were seeded on the prepared electrospun fibrous scaffolds, and the cytotoxicity of the fibers was examined using the MTT cytotoxicity assay [167]. The tested electrospun fibrous scaffolds were reported to be nontoxic, and cell viability depended on the amount of collagen used in their preparation. It was found that cell adhesion and cell growth were enhanced as the collagen percentage was increased.

**Figure 9:**
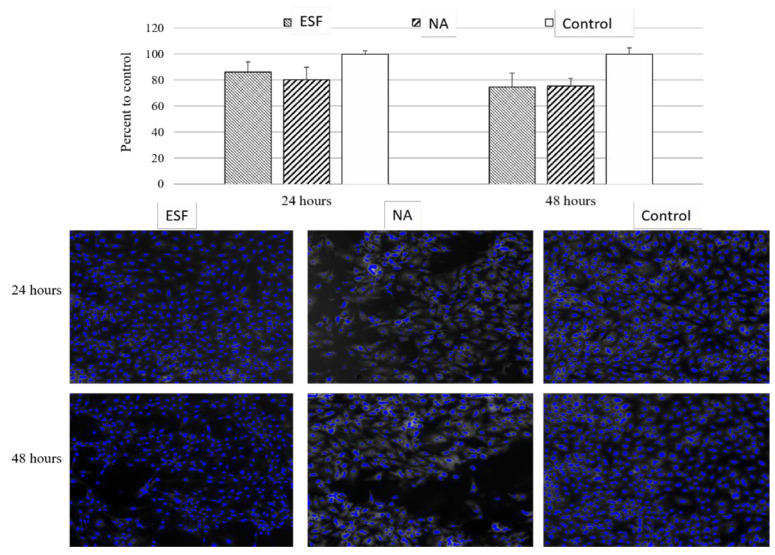
The effect of different synthesized scaffolds on H9C2 cell viability. Cells were incubated with both ESFs and NA for 24 and 48 h, and cell number was assessed by automated quantitation of DAPI-positive nuclei using ArrayScan XTI (target activation module). (**top**) The number of nuclei of viable cells is represented as percentages relative to untreated control. Data presented as mean ± SEOM, n = 6. Statistical significance: no statistical significant cytotoxicity detected compared to the control. (**bottom**) Representative images of the DAPI-stained nuclei of viable cells after incubation with the scaffolds (reproduced from reference [134] with permission).

**Figure 10:**
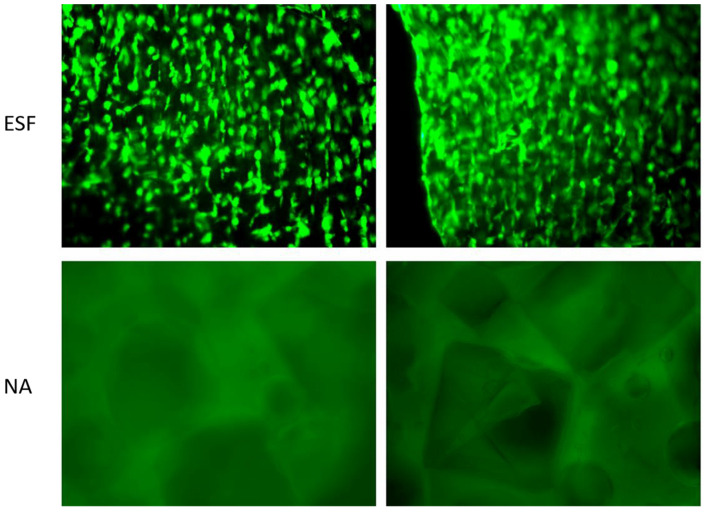
Qualitative assessment of cell/scaffold interaction in nontissue culture-treated plates. H9C2 cells were directly seeded on the scaffolds and incubated for 14 days in nontissue culture-treated plates. On day 14, cells were stained with Calcein-AM, and representative images of H9C2 cells on ESFs (**top**) and NA (**bottom**) were captured using a fluorescent microscope. Live cells appear as a fluorescent green color. (Reproduced from reference [134] with permission).

Another recent study was carried out using H9C2 cardiomyoblast cells to investigate the cytocompatibility of in situ UV poly (1–10, decandiol-co-tricarbllyate) elastomeric photo-crosslinked ESF in comparison with sodium chloride particulate-leached scaffolds (NA) of the same polymer for cardiac tissue engineering applications [134]. The sterile prepared ESFs were placed in ultra-low-attachment (non-tissue culture-treated) plates, and cells were added directly to the scaffolds and incubated for 14 days. At the end of the incubation period, viable cells attached to the scaffolds were stained using the Calcein-AM from the LIVE/DEAD^®^ viability/cytotoxicity assay. Cell viability was assessed visually by estimating cell-covered areas compared to the control. The results demonstrated no significant cytotoxicity after 24 or 48 h of incubation with either scaffold. Compared to the control, the viability was 86.2 ± 11.0% for ESFs and 80.4% ± 13.7 for sodium chloride particulate-leached scaffolds (NA). The data suggested that both scaffolds exhibited no significant cytotoxic effect in vitro (Figure 9); however, it was shown that H9C2 cardiomyoblasts successfully attached to ESFs, while there was no apparent attachment to the same polymeric scaffolds prepared using NA leaching. Compared to the NA scaffold, this higher degree of attachment on ESFs was attributed to optimum pore size, pore size distribution, and fiber diameter. In addition, H9C2 cells also demonstrated a certain degree of alignment along the ESF, potentially due to the fiber collection method used in the study, which resulted in offering the required anisotropic effect consistent with cardiac tissue engineering applications (Figure 10).

### 4.2. In Vitro Genotoxicity Assays and Gene Expression Alterations

The ESFs’ unique properties, including but not limited to nanometer-scale size, high surface area, variable chemical composition, surface structure, and shape, may allow them or their fragments to directly interact with biological systems and subsequently alter cell signaling and function. Many well-established techniques have been reported, which include the determination of gene mutations using the Ames assay in Salmonella typhimurium and *Escherichia coli* [168], the identification of DNA base modifications via measurement of oxidized guanine bases [169], and finally, the comet assay (single-cell gel electrophoresis) as a method for measuring deoxyribonucleic acid (DNA) strand breaks in eukaryotic cells [170].

Gene expression assays, also known as gene profiling, are also essential tools to assess gene expression alterations, including Northern blot analysis, ribonuclease protection assays (RPA) [171], quantitative real-time polymerase chain reaction (qRT-PCR) [172], PCR arrays, and microarrays [173].

For example, a recent study reported the fabrication of gelatin-based electrospun nanofibers crosslinked using horseradish peroxidase (HRP) gelatin for plasmid DNA (pDNA) delivery in tissue engineering applications [174]. The nanofibers were obtained through the electrospinning of an aqueous solution containing gelatin possessing phenolic hydroxyl moieties (Gelatin-Ph) and HRP with subsequent HRP-mediated crosslinking of the phenolic hydroxyl moieties by exposure to air containing H_2_O_2_. The Lipofectamine/plasmid pDNA complexes were immobilized on the nanofibers through immersion in the solution containing the pDNA complexes, resulting in transfection and sustained delivery of pDNA. To evaluate the validity of gene delivery from nanofibers, the HEK293EGIPneo cells stained with CytoRed were seeded on the same nanofiber mats, and transfection efficiency was measured using flow cytometry after 4 days of cell culture. The results indicated that immobilization of the pDNA complexes on the nanofibers inhibited their complexation with serum proteins to effectively deliver pDNA into cells [174].

### 4.3. In Vitro Degradation and In Vivo Animal Testing

The in vitro degradation behavior of electrospun fibers usually involves immersing the scaffolds typically in phosphate-buffered saline (PBS) solution of pH 7.4 at 37 °C for a predefined period. Degradation media selection and the buffer pH are determined based on the intended use of the fabricated ESF and the site of administration or implantation. Traditionally, to evaluate the degradation rate and extent with time, the following properties of the ESF are investigated: water uptake, pH buffer change, and relative weight loss [175]. In many studies, when the ESFs’ mechanical properties are significant, the ESF specimens are also subjected to mechanical testing to examine the changes in the mechanical parameters, including Young’s modulus, stress, and strain [60].

As with many other materials intended for biomedical applications, fabricated ESFs can also be subjected to in vivo animal studies to verify their effectiveness, efficacy, biological safety, and biocompatibility. This is mainly to test for any potential risks that can be caused by chemicals that are leached from the fibers and absorbed by the body, any possibility of immune or allergic reactions, in vivo degradation profiles, and the possibility of deviation from the intended growth into the body mainly for those ESFs intended for drug delivery and TE applications. With recent advances and big data intelligence, the future of biocompatibility testing might just be animal-free [176]. Wistar rats [177] and Sprague Dawley rats [51,178,179] are among the most common animals used to test the in vitro degradability or efficiency of drug-loaded fibers for tissue engineering and drug delivery. For more details on the in vivo evaluations, animal studies, and clinical trials conducted on electrospun nanofibers for biomedical applications, the reader is advised to refer to more focused reviews on this matter [180,181].

## 5. Regulatory, Environmental, and Safety Considerations

There is a discrepancy between different regulatory bodies worldwide in the definition, categorization, and evaluation of ESF-based matrices intended for various pharmaceutical and biomedical applications. The most common regulatory approach would categorize them into pharmaceuticals or medical devices. Although both categories are used to diagnose, cure, or prevent certain diseases, they are categorized as pharmaceuticals when nanofibrous matrices load an active chemical drug to exert a pharmacological effect on the human body. On the other hand, they are categorized as medical devices when they do not contain any active drug substance, so they do not achieve their purpose as a drug through chemical action [182]. As such, for example, tissue-containing nanofibrous-based matrices may be considered medical devices according to some jurisdictions but not according to others [183]. As pharmaceutical product development, premarket approval, and regulatory processes are well established worldwide, the following discussion will focus on approving ESF-based medical devices in the US market.

The Center for Devices and Radiological Health (CDRH) is the United States Food and Drug Administration (FDA) section responsible for the premarket approval and postmarketing monitoring of all medical devices and oversees their manufacturing, performance, and safety. Medical Device Amendments to the Federal Food, Drug, and Cosmetic Act (FD&C Act) were first issued in 1976 and continued evolving with subsequent laws until the year 2002 when the Medical Device User Fee and Modernization Act (MDUFMA) went into effect, followed by the Medical Devices Technical Corrections Act released on 1 April 2004 [184]. It is essential to mention that the fabricated ESF would be considered under a combination product definition when it involves at least two regulatory component types of a drug, device, or biologics (ex., drug-eluting fibrous-made cardiovascular stents). In this case, the regulatory responsibilities would stem from involved component types and would be facilitated and coordinated under the Office of Combination Products jurisdiction [185].

Based on device description and intended use, the FDA classifies devices into Classes I, II, and III, reflecting the extent of regulatory control and increasing with degree of risk. Class I typically includes devices with the lowest risk and are regulated only by general control. On the other hand, Class II medical devices with moderate risk require special and general controls. Class III devices are typically used to sustain human life and are of substantial importance in preventing impairment of human health. They possess the highest risk and require premarket approval (PMA) from the FDA to obtain marketing. The PMA is usually a scientific and regulatory review process to evaluate the safety and effectiveness of Class III medical devices, including a nonclinical laboratory studies section and a clinical investigations section. Similar to a drug approval process, the clinical evaluation for Class III devices, such as implantable or other high-risk devices, must be based on evidence gathered through clinical investigation. Moreover, clinical investigations must fulfill the Good Clinical Practice (GCP) requirements regarding data quality, integrity, and ethical standards. The premarket approval regulations are in the Title 21 Code of Federal Regulations (CFR) Part 814 [186]. Examples of FDA-approved electrospun-fiber-based medical devices are listed in Table 4. The process of securing approval for the devices passes through many stages regulated by the FDA and can be summarized in Table 5. One of the most critical aspects that must be taken into consideration in the early development of electrospun-fiber-based medical devices is the choice of polymers and solvents used in the electrospinning process. The best choice is to use FDA-approved biocompatible polymers (e.g., PLGA, PCL, lactides) and solvents that satisfy safety and environmental requirements with nontoxic or below-level residuals. Nonsolvent melt electrospinning would also be an ideal choice as it waives solvent utilization and ensures no toxicity with minimal effect on the environment. Another aspect of fabricating ESF-based medical devices that must be taken into consideration is the ability to conduct the fabrication process under good laboratory practices (GLP) during the development phase and the ability to conduct the fabrication in clean rooms, which ensures that both in-process and final product sterility requirements are met. This aspect is critical, as the later stages of regulatory approvals require in vitro and in vivo animal testing before advancing to the three phases of the clinical trials. Finally, manufacturing electrospun nanofibrous materials for clinical trials necessitates the availability of a Good Manufacturing Practice (GMP) and ISO 13485-certified development and manufacturing facility with capabilities for scale-up while meeting all regulatory requirements [187].

## 6. Conclusions and Future Perspectives

RES is mainly categorized into CRES and PRES. Each method has different crosslinking strategies and agents, which were thoroughly reported and examined in this review. The CRES technique is more straightforward for scaffold crosslinking and requires less initial polymer modification but consistently results in heterogeneous fibers with less compatibility due to the chemical toxicity of crosslinkers and harsh chemical solvents used. PRES, conversely, is considered less harsh, does not utilize toxic crosslinking agents, and results in uniform, homogenous, and more cytocompatible crosslinked fibers, which can be more challenging to achieve with some chemical crosslinking variants. Also, PRES is quicker, more effective, and better suited for manufacturing tissue engineering and drug delivery scaffolds on a large scale. In addition, it is simple to adjust the mechanical characteristics of scaffolds and their water stability to suit the needs of particular uses in PRES. One of the challenges facing PRES is the need to undergo an initial modification of the polymer material or the addition of photoresponsive polymers, which include additive initial reactions. This requires developing new green chemistry and purification techniques that maximize biocompatibility and reduce environmental impact.

Many challenges and research gaps are still being faced in the advancements of RES-based ESFs, particularly for drug delivery and TE applications. Large-scale fabrication and the need for industrialized GLP and GMP/ISO-certified facilities to advance the developed products into approval phases and commercial manufacturing are still needed.

Although the FDA approved a few ESF-based devices for TE applications, there is still a lack of clinical trials, as most studies are conducted in vitro or on animal models. The critical need for standardization of the electrospinning procedures, materials compatibilities, and ethical approval is imperative to transfer the product to a commercial market.

From a marketing and economic point of view, researchers involved in electrospinning, especially at universities, must start developing business plans and build ties with the relevant industry early enough to address possible fabrication and economic challenges accompanying emerging electrospinning technologies, which will be aggravated when the developed technology is directed towards clinical applications.

Finally, there is no doubt that advancements in AI and ML and their integration in many aspects of research and development will dilute many of the discussed challenges. The ongoing AI adoption for predictive toxicology, data analysis, risk assessment, and mechanistic research is resulting in the automation and standardization of many complex processes and fabrication technologies, including the emergence of promising animal-free toxicological studies needed in clinical trials. Undoubtedly, developing innovative electrospinning approaches like RES are necessary to keep the ES technology platform continuously offering fibers’ versatility and unique nanostructure features beyond any of the currently existing technologies.

## Figures and Tables

**Table 1:** A list of commonly used RES chemically synthesized and naturally occurring crosslinkers with their corresponding chemical structures and reported cytocompatibility.

Class		Crosslinker	Chemical Structure	Cytotoxicity Profile
Synthetic Crosslinkers	Carbodiimide	N-ethyl-N-(3-(dimethylamino) propyl) carbodiimide hydrochloride (EDC)	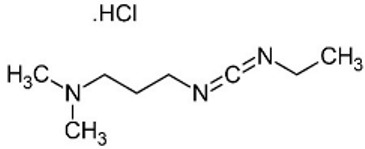	L929 cells were cultured on the gelatin scaffold with a concentration of 15 mM EDS: NHS in a ratio of 1:2. The results indicated successful cell attachment, growth, and proliferation over 7 days during the culture period [50].
		N-hydroxysuccinimide (NHS)	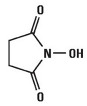	
	Aldehyde	Glutaraldehyde (GTA)	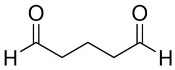	After 24 h exposure to GTA vapor, cell viability within gelatin fibers decreased to 70% after one week [60].
		Glyoxal	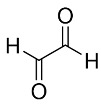	An amount of 5% (*w*/*w*) (40% aqueous solution) of glyoxal had no detrimental impact on the cytocompatibility of the nanofibers. Fibroblast cells demonstrated successful adhesion and proliferation on the mats’ surface for over 15 days [27].
	Epoxides	1,4-butanediol diglycidyl ether (BDDGE)	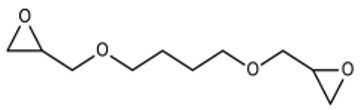	BDDGE crosslinkers at 2%, 4%, and 6% (wt/wt) were assessed for toxicity on fibroblast cells over 7 days. The results revealed no toxicity, as cells successfully attached and proliferated within the electrospun meshes [34].
	Isocyanate	1,6-hexamethylene diisocyanate	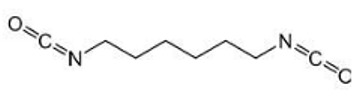	An increase in the percentage of isocyanate in dental resins was reported to cause cytotoxicity in human gingival fibroblast cells [73].
	Silane	Tetraethylorthosilicate (TEOS)	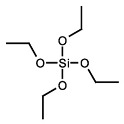	The fibers facilitated mammalian cell proliferation and growth at 0.1% (*w*/*w*) TEOS. At higher concentrations, a cytotoxic effect was observed [61].
	Hydrazide	Hydrazide	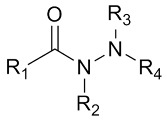	A thiolated hyaluronic acid derivative, 3,3’-Dithiobis (propionohydrazide)-modified HA (HA-DTPH), showed effective growth of NIH 3T3 fibroblasts on the scaffolds [26].
Natural Crosslinkers	Plant Extract	Genipin	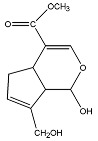	It can potentially reduce the immunogenicity of xenogeneic decellularized whole-liver scaffolds [74].
	Acids	Citric acid (CA)	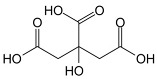	Citric acid 1% (*w*/*w*) concentration demonstrated nontoxicity in human fibroblast cells cultured for 72 h. Higher concentrations had a minor negative impact on fibroblast viability. [71].
		Phytic acid (PA)	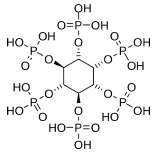	Scaffolds containing 7.5% (*w*/*w*) or lower PA content can achieve over 80% fibroblast cell viability across all concentrations [72].

**Table 2:** Representative list of recently reported research on utilizing CRES, PRES, and other RES techniques for TE applications with details on the polymers used, the RES technique utilized, the type of crosslinker or the photoinitiator used, and the photo radiation source.

Polymer	RES Type	Crosslinker/Radiation	Initiator	Applications	References
Hyaluronic acid	CRES	Thiolated HA derivative, 3,3′-dithiobis(propanoic dihydrazide)/poly(ethylene glycol) diacrylate	N/A	Wound healing/TE	[26]
Poly (2-hydroxyethyl methacrylate)/2-ethoxy ethyl methacrylate	CRES	Ethylene dimethacrylate	N/A	Biomedical application	[138]
Chitosan	CRES	Glutaraldehyde	N/A	TE	[139]
Poly (methyl methacrylate-co-2-hydroxyethyl acrylate)	PRES	Ultraviolet light	2,2′-azobis (isobutyronitrile)/no photoinitiator	Artificial extracellular matrix	[99]
Polyurethane/polyethylene glycol methacrylate	PRES	UV light	Benzophenone	Vascular TE	[116,117]
Poly (trimethylene carbonate-l-lactide)	PRES	Gamma radiation	Camphorquinone	TE	[110]
Polyamide 66	PRES	Gamma radiation	N/A	TE	[111]
N-octyl cyanoacrylate	CRES	Atmospheric water molecules/amine groups in the liver	N/A	TE	[127]
Poly (2,3 L-hydroxy carbonate)	PRES	UV	Bis (2,4,6trimethylbenzoyl) phenyl-phosphine oxide	Vascular TE	[100]
Medical polyurethane	PRES	UV	Cumene hydroperoxide (CHP), dicumyl peroxide (DCP)	Biomedical application	[121]
L-polyethylene imine	PRES	UV	Phenyl-bis(2,4,6-trimethyl benzoyl)-phosphine oxide	TE	[120]
Polydimethylsiloxane (PDMS)	TRES/CRES	100 °C heated collector	N/A	Tissue engineering	[75]
Poly(succinimide) (shell) and 2,2,4(2,4,4)-trimethyl-1,6-hexanediamine (core)	CRES	2,2,4(2,4,4)-trimethyl-1,6-hexane diamine	N/A	Tissue engineering	[29]
Poly (oligo-ethylene glycol methacrylate) (hydrazide-functionalized and aldehyde-functionalized)	CRES	Covalent crosslinking	N/A	Wound healing	[63]
Methacrylated Zein	PRES	UV	Phenyl-bis(2,4,6-trimethyl benzoyl)-phosphine oxide	Skin tissue engineering	[136]
Gelatin hydrogel	PRES	UV	Irgacure 2959	Tissue engineering	[101]
Acrylated polysulfone	PRES	UV	N/A	Tissue engineering	[140]
Fish collagen/polycaprolactone	CRES	1-ethyl-3-(3-dimethyl aminopropyl)carbodiimide, N-hydroxysuccinimide	N/A	Skin tissue engineering	[128]
ELR-clickable fiber	CRES	N/A	N/A	Dermal application	[129]
Poly(ethylene glycol) diacrylate (PEGDA)/gelatin	CRES	hexamethylene diisocyanate	Irgacure	Abdominal wound healing	[133]
	CRES	Glycoxal	N/A	Skin tissue engineering	[27]
Gelatin/polylactic acid	CRES	1-ethyl-3-(3-dimethyl aminopropyl)carbodiimide, N-hydroxysuccinimide	N/A	Biological analysis	[141]
Gelatin/hydroxyapatite	CRES	Glycoxal	N/A	Periodontal tissue engineering application	[135]
Gelatin methacryloyl/dopamine	PRES	N/A	/	Wound healing	[118]
Polyaniline, acrylic acid (AA), polyethylene glycol diacrylate, acrylamide	PRES	UV	2-hydroxy-2-methylpropiopheno	Soft actuators	[142]
Gelatin methacryloyl	PRES and CRES	UV/tannic acid	Irgacure 2959	Tympanic membrane regeneration	[143]
PVA/gelatin	CRES	Transglutaminase	N/A	Wound healing	[66]
Chitosan/polyethylene oxide/ZnO	PRES	UV	Pentaerythritol triacrylate	Antibacterial	[130]
Polyvinylpyrrolidone (PVP)	PRES	UV	Benzophenone	Antibacterial	[131]
D-phenylalanine (D-PHI)/polycarbonate polyurethane	PRES	UV	Irgacure 1173	Tissue engineering	[96]
Gelatin methacryloyl	PRES	UV	Irgacure 2959	Nerve tissue engineering	[137]
Collagen and polyethylene terephthalate	PRES	UV	Riboflavin	Tissue engineering	[97]
Polycaprolactone and functionalized gelatin	PRES	UV	Irgacure^®^2959	Skin tissue engineering	[102]
Polylactic acid/polyethylene glycol	PRES	UV	N/A	Tissue engineering	[107]
PCL/bisphenol A diglycidyl ether	PRES	UV	Bis(4-tert-butyl phenyl) iodonium hexafluorophosphate), (2,2-dimethoxy-2-phenyl-acetophenone)	Shape memory effect	[144]
Poly(N, N-dimethylacrylamide) (G(DMAA)) and poly(DMAA-stearyl acrylate-dodecyl acrylate) (G(DMAA-SA-DA))	PRES	N, N-methylene bis(acrylamide)	N, N-methylene bis(acrylamide)	Fabrics	[145]
Poly(lactic acid)/dextran	PRES	UV	Phenyl-bis(2,4,6-trimethyl benzoyl)-phosphine oxide	Tissue engineering	[122]
Gelatin/dextran-methacrylate	PRES and CRES	NA	Darocur 2959	Tissue engineering	[119]
Polyvinyl alcohol/sodium caseinate	CRES	Glutaraldehyde	N/A	Antibacterial property	[86]
PVA/AgNO_3_	CRES	Glutaraldehyde	N/A	Antibacterial	[132]
Gelatin	CRES	1-ethyl-3-(3 dimethyl aminopropyl) carbodiimide hydrochloride (EDC) and N-hydroxysuccinimide (NHS)	N/A	Biomedical application	[50]
Polyvinylidene fluoride/hexafluoropropylene	PRES	Gamma	N/A	Prosthetic aorta	[146]
Collagen	CRES	Genipin and glutaraldehyde	N/A	Tissue engineering	[147]
Chitosan/polyvinyl alcohol	CRES	Genipin	N/A	Tissue engineering	[69]
Polysaccharide/hyaluronic acid, lactose-modified chitosan (CTL), and polyethylene oxide	CRES	Genipin, glutaraldehyde, 1-ethyl-3-(3 dimethyl aminopropyl) carbodiimide hydrochloride (EDC), and N-hydroxysuccinimide (NHS) and thermal crosslinking	N/A	Wound healing	[148]
Gelatin/PCL	CRES	Phytic acid	N/A	Skin tissue engineering	[72]

**Table 3:** A list of recently reported research on utilizing CRES, PRES, and other RE techniques for drug delivery applications with details on the polymers used, the RES technique utilized, the type of crosslinker or the photoinitiator used, and the photo radiation source.

Polymer	RES Type	Crosslinker/Radiation	Initiator	References
Bis-maleimide-terminated Poly-L-Lactide/bis-furan-terminated Poly-D lactide	CRES	Spontaneous Diels–Alder coupling	N/A	[157]
Polycaprolactone	PRES	Gamma radiation	N/A	[112]
3,3′-dithiobis (propanoic dihydrazide)-modified HA (DTPH-HA)	CRES	Polyethylene glycol diacrylate	N/A	[26]
Poly(N-isopropylacrylamide-co-N-isopropylmethacrylamide) (P(NIPAAm-co-NIPMAAm))	PRES	UV	Irgacure 2959	[153]
Methacrylated gelatin	PRES and CRES	Ethylene glycol dimethacrylate	Lithium phenyl-2,4,6 trimethylbenzoylphosphinates	[155]
PVP/PVA	CRES	Glutaraldehyde	N/A	[158]
Gelatin/polycaprolactone	CRES	Glutaraldehyde	N/A	[126]
Polyvinyl alcohol/graphene	CRES and TRES	NA	N/A	[159]
Polyglobalide	PRES	UV	2,2 dimethoxy-2-phenyl acetophenone	[105]
Gelatin	CRES	Glutaraldehyde	N/A	[156]
Zein/polyvinylpyrrolidone	TRES and CRES	NA	N/A	[126]
Carboxymethylcellulose/polyethylene glycol	CRES	Butane tetracarboxylic acid	Sodium hypophosphite	[150]
Polyvinylpyrrolidone, polycaprolactone, or polyethersulfone	PRES	UV	Benzophenone	[160]
Polyurethane, polyethylene glycol, and poly(ethylene glycol) diacrylate (PEGDA)	PRES	Visible	Camphorquinone	[95]
Poly-l-lactide (PLLA) and gelatin	CRES	NA	GTA	[161]

**Table 4:** Examples of FDA-approved electrospun-fiber-based medical devices.

Trade Name	Company	Applications
Cerafix^®^ Dura Repair	Acera Surgical, St. Louis, MO, USA	Dural defects repair
Covera^®^ Vascular Covered Stent	Becton Dickinson, Franklin Lakes, NJ, USA	Vascular tissue engineering
PK Papyrus^®^ Stent Coating	Biotronik, New York, NY, USA	Vascular tissue engineering
EktoTherix^®^	Neotherix, York, UK	Wound tissue engineering
Restrata^®^ Wound Matrix	Acera Surgical, St. Louis, MO, USA	Soft tissue engineering
Artifascia^®^	Nurami Medical, London, UK	Dural defects repair
ReBOSSIS^®^	Orthorebith Co., Yokohama, Japan	Bone tissue engineering

**Table 5:** Summary of the FDA regulatory steps and stages to deliver a new medical product to market.

Stage	Regulatory Required Elements	Regulatory Federal Code
Establishment of registration.	Identify product (device) description. Identify purpose: ○ Intended use (usually broad). ○ Indications for use (more specific). ○ Duration of use. ○ Target patient population (age range; disease).	21 CFR Part 807
2. Verification and listing.	Verify that the product is a medical device. Manufacturers must list their devices with the FDA and provide information about them, such as contract manufacturers, contract sterilizers, specification developers, etc.	
3. Classification and regulatory pathway.	Identify the regulatory classification of the device (Class I, II, or III). Classification will generally indicate the regulatory pathway. Class III devices require premarket approval (PMA).	21 CFR Part 814
4. Valid scientific evidence.	Develop valid scientific evidence for safety and effectiveness. Define valid scientific evidence.	21 CFR 860.7(c)(1) 21 CFR 860.7(c)(2)
5. Preparation of the premarket submission (PMS).	Each type of submission needed depends on device class and has its own sets of processes, applicable laws and regulations, review times, and evidence burden. These types of PMS include the following: ○ *Investigational device exemption (IDE)*—clinical research on investigational device. ○ *Premarket notification (510(k))*—for low- and moderate-risk devices, it requires establishing a “Substantial Equivalence” between the new device and a legally marketed device employing intended use, device features, and performance testing. ○ *Premarket approval application (PMA)*—for Class III Devices; provide safety and effectiveness evidence on its own, i.e., not equivalent to any device. ○ De novo *classification request*—Provide a marketing pathway to classify novel medical devices for which general controls alone, or general and special controls, provide reasonable assurance of safety and effectiveness for the intended use but for which there is no legally marketed predicate device.	21 CFR Part 812 21 CFR Part 807

## Data Availability

All data are contained within the article.
